# Mindfulness-based programs for substance use disorders: a systematic review of manualized treatments

**DOI:** 10.1186/s13011-020-00293-3

**Published:** 2020-07-29

**Authors:** J. Richard Korecki, Frank J. Schwebel, Victoria R. Votaw, Katie Witkiewitz

**Affiliations:** 1 Department of Psychology, University of New Mexico, 2650 Yale Blvd SE, Ste. 200, Albuquerque, NM 87106 USA; 2 Center on Alcohol, Substance use, And Addictions (CASAA), University of New Mexico, Albuquerque, USA

**Keywords:** Mindfulness, Mindfulness-based interventions, Addictive behavior, Behavior change, Substance use disorders, Treatment

## Abstract

**Background:**

Substance use disorders are prevalent and returning to substance use (i.e., relapse) following treatment is common, underscoring the need for effective treatments that will help individuals maintain long-term reductions in substance use. Mindfulness-based interventions (MBIs) have been increasingly developed and evaluated for the treatment of substance use disorders. The aim of this article was to update a systematic review conducted by Li et al. in 2017 on the outcomes of randomized control trials of MBIs for substance use disorders. In addition, we provided a session-by-session examination of the most widely used MBI protocols.

**Methods:**

We conducted a comprehensive literature search of the PubMed, PsycINFO, and Web of Science databases from January of 2016 through April of 2020. Studies were included based on the following criteria: 1) examined the effects of an MBI, 2) employed a randomized controlled trial design with repeated measures, including secondary data analyses of randomized controlled trials, and 3) enrolled participants seeking treatment for substance use disorders.

**Results:**

The search identified 902 publications and 30 studies were eligible for inclusion and data extraction. MBIs appear to be as effective as existing evidence-based treatments for substance use disorders at reducing the frequency and quantity of alcohol and drug use, substance-related problems, craving for substance use, and at increasing the rate of abstinence.

**Conclusions:**

Future directions include additional large scale randomized controlled trials, investigation of the most suitable settings and protocols, examination of patient populations that may benefit most from MBIs, and dissemination and implementation research.

## Introduction

Substance use disorder (SUD) is a significant public health problem associated with considerable social and economic costs in the United States (U.S.) and throughout the world. According to the 2018 National Survey on Drug Use and Mental Health, approximately 20.3 million people ages 12 or older suffered from a SUD [1]. The estimated cost associated with substance use in the U.S. as measured by crime, lost work productivity, and health care is nearly $740 billion annually [2]. Furthermore, approximately 40–60% of individuals relapse within the first year following SUD treatment [3]. The large-scale prevalence of SUDs and the frequency of relapse after SUD treatment underscores the need to develop effective treatments that will help individuals maintain long-term changes in substance use.

A treatment option for promoting long-term health behavior change that has gained popularity in recent years is mindfulness-based intervention (MBI). One definition of mindfulness includes paying attention in the present moment in a particular way: on purpose and without judgment [4]. It is experiencing the true nature of the moment at hand without the biases created from past experiences, or the expectations of future events. The practice of mindfulness meditation has been performed for thousands of years and was traditionally taught to reduce the suffering of the human experience and to cultivate well-being [5]. Over the last few decades, secularized meditation practices have been integrated into several more traditional Western healthcare settings.

Mindfulness-Based Stress Reduction (MBSR), developed in the early 1980s, was the first mindfulness-based practice introduced into Western health care settings [6, 7]. MBSR was initially developed for patients with chronic pain and early trials identified reductions in measures of pain, negative body image, mood disturbance, anxiety, and depression following MBSR [6–8]. Since the success of MBSR in the early 1980s, there have been several MBIs developed to treat a multitude of conditions as both standalone treatments and adjuncts to already established treatments. Such interventions include Mindfulness-Based Cognitive Therapy (MBCT) [9], Mindfulness-Based Eating, Mindfulness-Based Childbirth and Parenting [10], and other third-wave therapies that include aspects of mindfulness, such as Dialectical Behavior Therapy (DBT) [11] and Acceptance and Commitment Therapy (ACT) [12].

Several MBIs specifically designed for SUD have been developed such as Mindfulness-Based Relapse Prevention (MBRP) [13, 14], Mindfulness Oriented Recovery Enhancement (MORE) [15], Mindfulness Training for Smokers (MTS) [16], Moment-by-Moment in Women’s Recovery (MMWR) [17], and other interventions designed to address factors theorized to maintain SUD. Craving is one such factor that is theorized to maintain SUD and targeted by MBIs. One of the major facets of MBIs for SUD is a non-judgmental observation of thoughts and behavioral urges. By divorcing oneself from the feelings associated with the craving (i.e. desire, aversion, physical or emotional discomfort, etc.), an individual may be able to “ride out” cravings without engaging in substance use. Another common aspect of addictive behaviors targeted by MBIs is the automaticity of action (i.e. “acting on autopilot” [18]). For example, MBIs target substance use as an automatic reaction when exposed to feelings, locations, and people that serve as cues for substance use. Mitigating automatic behaviors by bringing purposeful attention to the present moment may help to reduce the frequency and quantity of substance use and substance-related problems.

In recent years, several reviews and meta-analyses have investigated the efficacy of MBIs for the treatment of SUD. Li and colleagues [19] conducted a systematic review and meta-analysis of 42 studies that examined the effects of various MBIs on substance use. Findings from this review indicated that MBIs were more effective than control conditions (e.g., treatment as usual (TAU), relapse prevention treatment (RP), cognitive behavioral therapy (CBT), support group) at reducing the frequency and amount of substance use, the number of substance-related problems, level of craving for substance use, and at increasing rates of abstinence [19]. A 2018 meta-analysis of MBIs for SUD conducted by Goldberg and colleagues [20] found that MBIs were generally equivalent to evidence-based conditions and superior to other control conditions (e.g., minimal treatment, non-specific active controls). However, the efficacy of MBIs, as compared to control conditions, differed by follow-up period (i.e., post-treatment versus longer-term follow-ups) and targeted disorder. These mixed results highlight the need for larger, randomized clinical trials and an understanding of the subgroups of participants that may respond best to MBIs. With the emergence of varied MBIs for SUD, there is a need for more research into the most effective duration, meditation techniques taught, settings, and potential participants for these mindfulness-based programs.

The purpose of this manuscript was twofold. First, we reviewed treatment protocols of MBIs for SUD. Second, we conducted a systematic review of randomized controlled trials (RCTs) of MBIs for SUD, with a focus on studies published after 2017 to update the systematic review conducted by Li et al. Specifically, we aimed to provide a detailed account (session-by-session outline) of the most frequently studied MBIs, followed by a description of the current outcomes of those interventions based on findings of the systematic review. We then synthesized information gathered from the systematic review to address clinical implications, current limitations in the field, and suggestions for the road ahead.

## Method

### Search strategy

Given our aim of updating and extending the Li et al. systematic review published in 2017, our search strategy and guidelines for study eligibility were guided by this prior manuscript. We searched PubMed, PsycINFO, and Web of Science databases from January of 2016 through April of 2020. Search terms included the following combinations: (‘mindfulness’ OR ‘mindfulness intervention’ OR ‘mindfulness meditation’ OR ‘mindfulness treatment’ OR ‘mindfulness-based relapse prevention’ OR ‘mindfulness-based stress reduction’) AND (‘substance use’ OR ‘alcohol’ OR ‘cocaine’ OR ‘opioid’ OR ‘tobacco’ OR ‘marijuana’ OR ‘drug’). We also reviewed the reference list of eligible articles to identify additional studies not identified in our initial search. All methods were carried out in accordance with the Preferred Reporting Items for Systematic Reviews and Meta-Analyses (PRISMA) guidelines [21]. This systematic review was not pre-registered prior to publication.

### Study selection and data extraction

Peer-reviewed manuscripts published from January of 2016 to April of 2020 were included based on the following criteria: 1) examined the effects of an MBI, 2) employed an RCT design with repeated measures, including secondary data analyses of RCTs, and 3) enrolled participants seeking treatment for SUD. Studies were excluded for the following reasons: 1) non-peer reviewed publications, case reports, case series, editorials, commentaries, letters to the editor, book chapters, previously published narrative reviews, theses/dissertations, and study protocols, 2) only reported qualitative results, 3) non-randomized trials without a control group, 4) examined very brief mindfulness interventions (due to a lack of structured, manualized protocols and intervention content that varied from study to study), 5) did not assess substance use outcomes, 6) examined interventions without a formal mindfulness component (e.g., Acceptance and Commitment Therapy, Dialectic Behavior Therapy, etc.) or without a specific focus on SUD (e.g., Vipassana).

The first author (JRK) conducted the initial search. Authors JRK, FJS, and VRV determined eligibility, addressed eligibility questions, and performed data extraction. Data extraction was performed using a Microsoft Excel template to collect the following information (if available): citation (i.e., first author, year, journal); study aims; total sample size and sample size in the experimental and control conditions; overview of the population (e.g., demographics, relevant recruitment methods); description of the treatment and control conditions; targeted behavior; duration of treatment and timing of follow-up assessments; treatment compliance and attrition; additional notes on methodology; overview of results; and notes/limitations.

## Results

**Fig. 1:**
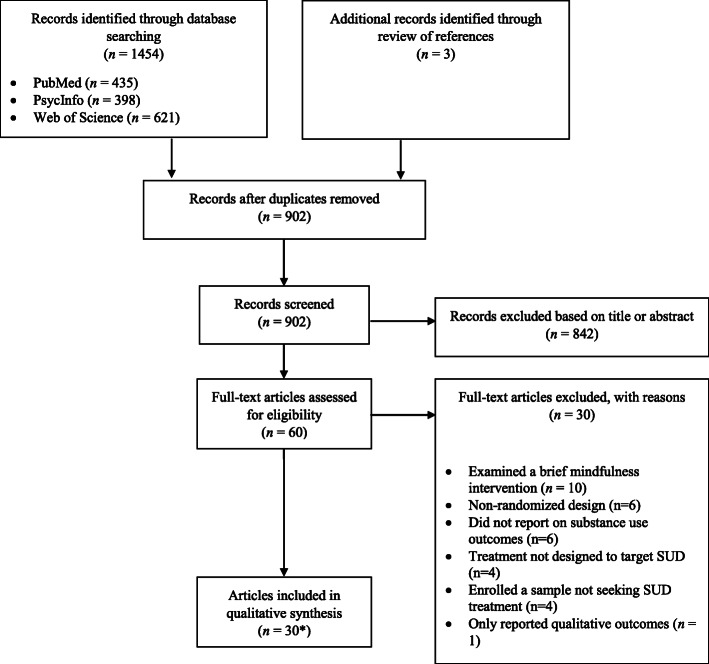
Flow diagram of records identified, screened, and included. *Note:* Records could be excluded for multiple reasons. *Includes one relevant erratum of an eligible manuscript

Results of the search are presented in Fig. 1. Our search identified 902 publications, of which, 60 full-text articles were assessed for eligibility. Thirty of the 60 articles were excluded for the following reasons: examined a brief mindfulness intervention (*n* = 10), employed a non-randomized design (*n* = 6), did not report on substance use outcomes (n = 6), active treatment not designed to target SUD (*n* = 4), enrolled a sample not seeking SUD treatment (n = 4), and only reported qualitative outcomes (*n* = 1); articles could be excluded for multiple reasons. Overall, 30 studies and one relevant erratum of an eligible study were eligible for inclusion and data extraction. A session-by-session outline of themes, primary practices, mechanisms studied, and moderators investigated for each of the six manualized protocols is presented in Table 1. Characteristics of included studies are presented in Table 2.

**Table 1:** Overview of treatment protocols

Intervention	Number of Sessions and length of the Protocol	Session 1 (Themes and Practices Taught)	Session 2 (Themes and Practices Taught)	Session 3 (Themes and Practices Taught)	Session 4 (Themes and Practices Taught)	Session 5 (Themes and Practices Taught)	Session 6 (Themes and Practices Taught)	Session 7 (Themes and Practices Taught)	Session 8 (Themes and Practices Taught)	Session 9 (Themes and Practices Taught)	Session 10 (Themes and Practices Taught)	Mechanisms Investigated	Moderators Investigated
Mindfulness Based Relapse Prevention (MBRP) [13]	8, 120-min group sessions	Automatic Pilot and Relapse. Primary practices: raisin exercise (mindful interaction with an ordinary object) and body scan meditation.	Awareness of Triggers and Craving. Primary practices: urge surfing and grounding meditation.	Mindfulness in Daily Life. Primary practices: mindfulness of breath and Stop Observe Breathe Expand Respond (SOBER space) introduction.	Mindfulness in High-Risk Situations. Primary practices: open awareness and walking meditation.	Acceptance and Skillful Action. Primary practices: SOBER space in difficult situations and mindful movement.	Seeing Thoughts as Thoughts. Primary practice: acknowledgment of thinking meditation.	Self-Care and Lifestyle Balance. Primary practice: loving-kindness meditation.	Social Support and Continuing Practice. Primary practice: concluding meditation (planting the seeds for continued mindfulness)	N/A	N/A	Impulsivity (+), craving (+), stress reactivity (+)	Anxiety (+), depression (+), gender (−)
Mindfulness Oriented Recovery Enhancement (MORE) [22]	10, 120-min group sessions	Mindfulness and the Automatic Habit of Addiction. Primary practice: chocolate exercise (mindful interaction with an ordinary object) and mindfulness of breath meditation.	Mindful Reappraisal. Primary practice: the power of reappraisal.	Focus on Savoring. Primary practice: mindful savoring (open awareness meditation).	Seeing Through the Illusion of Craving. Primary practice: chocolate exercise (breaking down the elements of craving and noticing the fleeting nature of urges).	Overcoming Craving and Coping with Stress. Primary practices: stress exposure and relaxation exercise and body scan meditation.	Attachment, Aversion, and Acceptance. Primary practices: thought suppression exercise and acceptance of cravings.	The Impermanent Body. Primary practices: impermanent body meditation and walking meditation.	Relationships and Relapse. Primary practice: loving-kindness meditation.	Interdependence and Finding Meaning. Primary practice: interdependence meditation.	Recovery and the Road Ahead. Primary practice: visualization of future self exercise.	Craving (+), cue reactivity (+), pain intensity (+)	Attentional bias (+), affective state (+), late positive potential (+)
Mindful Awareness in Body-Oriented Therapy (MABT) [23, 24]	8, 90-min group sessions	Identify Body Sensations. Primary practice: body literacy.	Articulate Body Sensations. Primary practice: self-massage.	Identifying and Attending to Internal Sensations. Primary practice: mindfulness of breath meditation.	Softening of Tension. Primary practices: tissue softening exercise and internal body attention exercise.	Sustained Mindful Attention. Primary practice: maintaining awareness on specific areas of the body.	Shift in Understanding. Primary practice: noticing internal shifts.	Reappraisal. Primary practice: reappraisal based on experiential awareness.	Interoceptive Awareness and Everyday Life. Primary practice: embodied self- awareness.	N/A	N/A	Interoceptive awareness (+), mindfulness skills (−)	Symptomatic distress (−)
Mindfulness-Based Addiction Treatment (MBAT) [25, 26]	8, 120-min group sessions	Automatic Pilot. Primary practice: breathing space.	Barriers to Mindfulness. Primary practice: body scan.	Mindful Breathing. Primary practice: mindfulness of the breath.	The Arising and Passing Away of Thoughts. Primary practice: traditional sitting meditation.	Being With What Is. Scheduled “quit day.” Primary practice: mindful movement (e.g., yoga)	Thoughts Are Just Thoughts. Primary practice: open awareness.	Taking Care of Yourself. Primary practice: integrating pleasant activities into everyday life.	Maintaining Mindfulness Going Forward. Primary practice: continue to use the skills learned in future situations.	N/A	N/A	Tobacco dependence (+), withdrawal (−), craving (+)	Negative affect (+), stress (+)
Mindfulness Training for Smoking Cessation (MTS) [16, 27]	8, 90-min group sessions	Habituation. Primary practice: introduction to mindfulness	Awareness of Triggers and Craving. Primary practice: Recognize, Accept, Investigate, and Note (RAIN) what cravings feel like as they arise practice.	Working with Stress and Negative Emotions. Primary practice: loving-kindness meditation.	Coping with Craving and Committing to Quit (“Quit Day”). Primary practice: urge surfing.	Mindfulness in Everyday Life. Primary practices: awareness of breath and walking meditation.	Automatic Pilot. Primary practice: noting meditation.	Acceptance. Primary practice: how our current thoughts and beliefs plant the seeds for future emotional states.	Course Summary. Primary practice: how to continue down the path of mindfulness.	N/A	N/A	Craving (+), abstinence from tobacco (+)	Stress reactivity (+), positive/negative affect (−)
Moment-by- Moment in Women’s Recovery (MMWR) [17, 28, 29]	8, 90-min group sessions and 1, 4-h “retreat day”	Introduction to Mindfulness. Primary practice: meditation of triggers.	Bringing Awareness to Thoughts, Emotions, Body Sensations, and Actions. Primary practices: mindful eating practice and standing body scan.	The Role of Perceptions in Relapse. Primary practice: noting practice.	Using Mindfulness to Deal with Negative Emotions. Primary practice: loving-kindness meditation.	The Role of Guilt and Shame in Relapse. Primary practice: mindfulness of painful thoughts and emotions.	Mindful Communication. Primary practice: difficult communication meditation.	Retreat Day. Primary practices: a compilation of all of the formal practices taught throughout the course.	Anger, Self- Violence, and Violence Towards Others. Primary practices: warning signs of anger and loving-kindness meditation.	Summary and Farewell. Primary practices: closing ritual and graduation ceremony.	N/A	Yes; Mindfulness (+), craving (+)	Yes; Affect (−), emotion regulation and distress tolerance (+)
Note: (+) = effect found, (−) = effect not found

**Table 2:** Characteristics of Studies Included in Systematic Review

Reference	Treatment condition	Control condition(s)	Overview of sample	Data collection time points	Outcome measures	Results	Limitations
Mindfulness-Based Relapse Prevention (MBRP)							
Abed et al., 2019 [30]	MBRP + Methadone Maintenance Therapy (MMT): 8, 120-min sessions.	No intervention control + MMT.	60 adult Iranian males (MRBP - 30 participants (26 completed), Control −30 participants (29 completed)) undergoing MMT in Isfahan, Iran. Participants were between the ages of 27 and 50 who had undergone MMT within the six months to one year prior to the beginning of the study. Prior heroin use ranged from two to seven years.	Baseline and post-intervention testing of the Heroin Craving Questionnaire (HCQ), and a urinalysis the first, second, and third months after the eight-week intervention.	Primary outcomes were differences among the scores of craving and desire (HCQ) and lapse occurrence (drug urine tests).	The mean of the post-test scores in the subscales of desire to use on the HCQ (pre-test M = 34.27; post-test M = 18.93), intention to use (pre-test M = 30.66; post-test M = 18.42) and anticipation of relief from withdrawal or dysphoria (pre-test M = 38.58; post-test M = 21.66) decreased from pre-test to post-test in the MBRP group. The results revealed a significant difference among the scores in the subscales of craving and the difference in the three subscales of desire to use, intention to use, and anticipation of relief from withdrawal or dysphoria was statistically significant. There was a marked reduction in positive drug tests for the MBRP group (9, 7, 14%) compared with the control (23, 24, 22%) over the three-month follow-up window.	There is a potential lack of generalizability in the study. The population was comprised of only male MMT patients. Without an active control group, it is hard to determine the effects of MBRP vs. the MMT.
Brown et al., 2020 [31]	Rolling MBRP + tDCS: 8, 120-min sessions with the first 30-min consisting of tDCS.	Rolling MBRP + sham tDCS: 8, 120-min sessions with the first 30-min consisting of the sham tDCS.	68 adults interested in reducing their drinking (Active tDCS = 36; sham tDCS = 32). 52.9% male, 52.16 (13.6) years old, 50.0% non-Hispanic white. 98.5% met DSM-5 criteria for current AUD with the remaining participant meeting criteria for lifetime AUD.	Baseline and post-intervention.	Alcohol cue-related hypersensitivity: EEG was recorded to capture an event-related component shown to relate to emotionally salient stimuli. The late positive potential (LPP), and self-reported craving and negative affect were recorded during an image presentation task.	There was a main effect of time in predicting craving ratings and LPP amplitudes for the alcohol images, such that craving ratings and LPP amplitudes significantly decreased over time. Also, there was an effect of group attendance, such that more groups attended was associated with lower craving rations. There was no effect of active versus sham tDCS in predicting craving ratings. Significantly higher LPP amplitudes were associated with the active tDCS compared with the sham tDCS.	The retention for the EEG assessment from the baseline to the post- treatment follow-up was low (54.4% completed both assessments).
Carroll et al., 2018 [32]	MBRP: 8, 120-min sessions.	Relapse prevention (RP): 8, 120-min sessions. Topics included self-efficacy, coping skills, goal setting, problem solving, and social support. TAU: process-oriented groups, such as 12-step, as facilitated regularly at the community treatment agency.	34 patients (MBRP = 12; RP = 12; TAU = 10) from a private, non-profit substance abuse care facility in the Pacific Northwest. 43.4 (9.7) years old, 73% male, and 47% non-Hispanic White.	Post-intervention (within the 2 months following completion of the larger RCT intervention).	The primary outcome was tonic and phasic heart rate variability (HRV) to a cognitive stressor (Electrocardiography (ECG)). Secondary outcomes included self- reported anxiety and craving during the cognitive stressor.	Prior to performing the stressor task, MBRP evinced greater tonic HF-HRV than RP, HF-HRV did not significantly differ between RP and TAU. All of the pairwise comparisons were significant (TAU vs RP, *p* = 0.013; TAU vs MBRP, *p* < 0.001; RP vs MBRP, *p* = 0.011) for phasic HRV. State anxiety increased during the stressor and quickly subsided during recovery in all three treatment groups. Groups did not differ significantly in baseline or recovery values, nor did they differ in rates of return to baseline. Group differences in baseline, recovery, or rates of recovery for craving were not observed.	This laboratory assessment was conducted as an extension of a large- scale clinical trial, and in consideration of participant burden, there is only one assessment time point with a relatively small sample.
Davis et at., 2018 [33]	Rolling MBRP + treatment normally provided by the residential facility: 8, 90- min classes that were held twice weekly. Members were enrolled as they entered the residential facility (as opposed to the standard 8- week cohort-based protocol). The basic treatment practice employed at the residential treatment center was a mix of cognitive-behavioral treatment and 12-step approach to recovery.	TAU + treatment normally provided by the residential facility: 8, 90-min sessions held twice weekly that were social support groups (Alcoholics and Narcotics Anonymous).	79 young adults (MBRP = 44; TAU = 35) enrolled in a residential SUD treatment facility. 25.3 (2.7) years old, the majority of clients were non-Hispanic White (91%), male (65%), and had less than a high school education (mean years of education was 11.9). Over 90% of participants were polysubstance users, and average length of stay at the treatment facility was 41 (26.2) days	Baseline, bi-weekly (every 2 weeks) assessments during treatment, 1- and 6- month post-treatment assessments.	Primary outcomes were perceived stress (PSS), craving (GAIN assessment), and substance use (SFS).	Those assigned to the TAU group tended to show immediate increases in substance use that peaked and subsequently plateaued around 13 weeks after treatment. The MBRP group’s substance-use trajectories differed markedly. Participants reported statistically significant declines in their level of craving during the treatment phase. These declines were statistically identical across treatment conditions. Those assigned to the MBRP condition largely maintained their low levels of craving throughout the remainder of the 28- week study period, those assigned to the TAU condition showed rather immediate and substantial increases in their craving levels, before plateauing approximately 14 weeks post-treatment. Participants in MBPR showed statistically significant improvements in their stress levels during the 8-week intervention.	Small sample size, but this is the first study to provide evidence and support for the use of MBRP among high risk, marginalized young adults in residential substance use disorder treatment.
Davis et at., 2019 [34]	Rolling MBRP + treatment normally provided by the residential facility: 8, 90- min classes that were held twice weekly. Members were enrolled as they entered the residential facility (as opposed to the standard 8- week cohort-based protocol). The treatment normally provided was a combination of cognitive-behavioral treatment and the 12-step approach to recovery.	TAU + treatment normally provided by the residential facility: 8, 90-min sessions held twice weekly that were social support groups (Alcoholics and Narcotics Anonymous).	79 young adults (MBRP = 44; TAU = 35) enrolled in a residential SUD treatment facility. 25.3 (2.7) years old, the majority of clients were non-Hispanic White (91%), male (65%), and had less than a high school education (mean years of education was 11.9). Over 90% of participants were polysubstance users, and average length of stay at the treatment facility was 41 (26.2) days.	Baseline, bi-weekly (every 2 weeks) assessments during treatment, 1- and 6- month post-treatment assessments.	Primary outcomes were impulsivity (UPPS-P impulsive behavior scale) and substance use (SFS).	Participants receiving MBRP evidenced significant reductions in all facets of impulsivity except for sensation seeking during treatment, and these reductions were significantly greater than TAU for all facets except positive urgency. These treatment gains were maintained through the 6-month follow-up.	The MBRP intervention in the current study made use of a rolling group admission, allowing new patients at the residential facility to enter the group and departing patients to leave it. This may be a limitation because patients could receive instruction out of order, weakening the integrity of the treatment.
Glasner-Edwards et al., 2017 [35]	MBRP + contingency management (CM): 8, 75- min sessions. Modified sessions from 120 min to increase engagement by shortening meditation practices. All participants in the trial also received CM, which consisted of twice-weekly visits of the fishbowl method, with rewards based on urine drug screes.	Health education (HE) + contingency management (CM): 8, 75-min sessions. Addressed 6 different types of health (e.g., intellectual, social, emotional, physical, environmental, spiritual). All participants in this condition also received twice-weekly CM visits.	63 adults (MBRP + CM = 31; HE + CM = 32) with DSM-IV stimulant dependence recruited from the community. 71.4% male, 45.3 (8.9) years old, 30.2% non-Hispanic white, 43% with a co-occurring Axis 1 disorder.	Baseline and 1- month post-treatment follow- up.	Substance use outcomes were stimulant-positive urine drug screens and the ASI addiction severity score. Psychiatric severity outcomes were the BDI, BAI, and ASI psychiatric severity score. Hypothesized mediators were emotion regulation (DERS), thought suppression (WBSI), and mindfulness skills (FFMQ).	There was no effect of treatment condition on odds of producing a stimulant-positive urine drug screen over the 8-week intervention phase (73% in MBRP + CM vs. 70% in HE + CM) or change in ASI addiction severity index scores from baseline to the 1-month follow-up. However, those with co-occurring psychiatric disorders had lower odds of producing a stimulant-positive urine drug screen if they received MBRP + CM vs. HE + CM. MBRP + CM was associated with greater decreases in BDI scores and increases in ASI psychiatric severity scores, but not BAI scores, as compared to HE + CM. There was no significant group x time interaction for any of the putative mechanisms of change (DERS, WBSI, FFMQ) over the study period.	Short follow-up. High attrition rates (23 of the 63 participants terminated study involvement). Shortened MBRP protocol.
Hsiao et al., 2019 [36]	MBRP: 8, 120-min sessions	TAU: 90-min sessions 1- 2x/week during the intervention phase. Twelve-step, process-oriented format.	Adults (study 1 *n* = 168, study 2 *n* = 198) recruited from an outpatient SUD treatment program who had completed inpatient or intensive outpatient treatment in the previous two weeks. Study 1: 63.7% male, 40.5 (10.3) years old, 53.6% non-Hispanic white. Study 2: 75.1% male, 38.2 (10.9) years old, 52.8% non-Hispanic white.	Study 1: Baseline, post-treatment, 2- and 4-month follow-up Study 2: Baseline, post-treatment, 2-, 4-, 6-, and 12- month follow-up.	Substance use outcome was craving (PACS). Examined a latent mindfulness factor as a mediator (AAQ and acting with awareness and nonjudgment subscales of the FFMQ).	Study 1: The effects of MBRP, as compared to TAU, on AAQ, FFMQ, and PACS scores at post-treatment were small-to-medium (Cohen’s d range from 0.08 to 0.48). The latent mindfulness factor significantly mediated the effects of MBRP, as compared to TAU, on lower craving scores post-treatment. Study 2: The effects of treatment condition on AAQ, FFMQ, and PACS scores at post-treatment were very small (Cohen’s d range from 0.03 to 0.21) and indicated that those who received TAU reported higher mindfulness scores than those who received MBRP. Higher post-treatment mindfulness was associated with lower post-treatment craving, but those in the MBRP condition did not have greater post-treatment mindfulness scores, and therefore there was not significant mediation.	Low reliability of the AAQ. Study 2 was conducted several years after study 1 from the same treatment program, and therefore the treatment program might have integrated components of MBIs in TAU, mitigating the effect of MBRP. The latent mindfulness factor was not invariant across the two samples, indicating that the measurement of mindfulness was not equivalent across studies.
Greenfield et al., 2018 [37]	MBRP: 8, 120-min sessions	Relapse prevention (RP): 8, 120-min sessions. Topics included self-efficacy, coping skills, goal setting, problem solving, and social support.	191 adults with SUD who were recruited following inpatient or intensive outpatient treatment. 71.0% male, 39.04 (10.93) years old, 52.9% non-Hispanic white. 43.5% of participants were in groups comprised of > 50% non-Hispanic white participants and 56.5% were in the groups comprised of > 50% racial/ethnic minority participants.	Baseline and 12- month post-treatment follow- up.	Number of drug use days and number of heavy drinking days in the 90-day period before the 12- month post-treatment follow-up.	Among racial/ethnic minority participants, there was not a significant difference in heavy drinking days between MBRP and RP, but those who received MBRP reported fewer drug use days than those who received RP. Among non-Hispanic white participants, those who received MBRP reported fewer heavy drinking days, as compared to those who received RP, but there was no difference in drug use days by treatment condition. Among individuals in groups comprised of > 50% racial/ethnic minority participants, there was not a significant difference in heavy drinking days between MBRP and RP. Among those in groups with > 50% non-Hispanic white participants, those who received MBRP had fewer heavy drinking days than those who received RP. There was not a significant interaction between group racial/ethnic composition and treatment condition in predicting drug use days. In subgroup analyses of only racial/ethnic minority individuals, there was a significant interaction between group race/ethnicity composition and treatment condition in predicting heavy drinking days, but not drug use days. Among racial/ethnic minority individuals in groups comprised of > 50% racial/ethnic minorities, MBRP produced fewer heavy drinking days than RP. There was no difference in heavy drinking days by treatment condition for racial/ethnic minority individuals who were in groups with > 50% whites. In subgroup analyses of only non-Hispanic white	Not able to examine particular racial/ethnic minority groups or differences by acculturation/racial identity.
Roos et al., 2017 [38]	MBRP: 8, 120-min sessions.	TAU: 12-step facilitation and process-oriented groups. Met 1-2x/wk. for 1.5 h. RP: (study 1 only) 8, 120-min group sessions. Treatment match control for MBRP	454 adults (study 1 *n* = 286, study 2 *n* = 168) recruited from an outpatient SUD treatment program who had completed inpatient or intensive outpatient treatment in the previous two weeks. Study 1: 71.8% male, 38.4 (10.9) years old, 51.6% non-Hispanic white. Study 2: 63.7% male, 40.5 (10.3) years old, 53.6% non-Hispanic white.	Study 1: Baseline, post-treatment, 3-, 6-, 12-month follow-up Study 2: Baseline, post-treatment, 2- and 4-month follow-up.	Alcohol and drug use days were assessed using the TLFB. Substance use disorder symptom severity was measured using the Severity and Dependence Scale (SDS) and the Short Inventory of Problems (SIP). Depression and anxiety symptoms were measured using the BDI-II and BAI, respectively	Study 1: Individuals with high SUD severity and high depression/anxiety and in the MBRP condition experienced significantly fewer heavy drinking days (HDD) and drug use days (DUD) at 12-month follow-up relative to RP and TAU. No effects were seen in low SUD severity and low depression/anxiety class. High SUD severity and low depression/anxiety and MBRP condition predicted significantly fewer 12-month HDD relative to RP or TAU. Study 2: MBRP condition for high severity and high depression/anxiety and high severity SUD and low depression/anxiety had significantly fewer alcohol and other drug use days relative to TAU.	A retrospective self- report measure of days abstinent was used, which may not accurately reflect actual alcohol and drug use days.
Roos et al., 2019 [39]	MBRP: 8, 120-min sessions	Relapse prevention (RP): 8, 120-min sessions. Topics included self-efficacy, coping skills, goal setting, problem solving, and social support.	117 adults recruited from an outpatient SUD treatment program who had completed inpatient or intensive outpatient treatment in the previous two weeks. 70.9% male, 53.8% non-Hispanic white, 39.0(10.9) years old.	12-month follow- up	Substance use was measured using the TLFB. Severity of substance use disorder was measured using the Severity and Dependence Scale.	Condition predicted fewer drug use days (DUD) such that MBRP participants reported 84% fewer DUD than RP participants. Individual gender and gender group composition not a significant predictor of heavy drinking days (HDD). There was a significant interaction between treatment condition and gender composition on DUD at 12- month follow-up such that MBRP participants had fewer 12-month DUD than those receiving RP and was most pronounced among groups comprised of one-third or more women.	Only a single follow-up 12-months after the completion of the intervention.
Shorey et al., 2016 [40]	MBRP + Acceptance and Commitment Therapy (ACT): 8 bi-weekly, 90 min sessions. 20–30 min of guided meditation followed by experiential activities and discussion. Daily homework recordings with a CD player. Attended when normally would have attended process groups.	TAU: Program guided by a 12- step model. Varied daily therapeutic activities including: process groups, AA/NA meetings, coping skills groups, family therapy, exercise groups, acupuncture, individual session with therapists	117 adults (MBI = 64; TAU = 53) in residential substance use disorder treatment (28–30 day program). 74.3% male, 92.2% non-Hispanic White, 41.3 (10.7) years old.	Baseline and post-treatment (at discharge)	Primary outcomes were: craving (PACS), mindfulness (FFMQ), and psychological flexibility (AAQ-SA).	No significant difference between conditions at discharge. Effect sizes between groups were small. MBRP + ACT participants reported lower craving and higher psychological flexibility.	Participants only attended an average of 5.4 of the 8 classes in the MBRP + ACT group. Attendance was not tracked in the TAU condition.
Weiss de Souza et al., 2020 [41]	MBRP + The Brazilian Ministry of Health’s tobacco cessation standard treatment (ST) protocol: 8, 120- min sessions. The ST protocol was a cognitive- behavioral treatment delivered in four structured weekly sessions and six maintenance follow-up sessions. Sessions were designed to provide information on risks of smoking and benefits of quitting, stimulate self- control and self-management to disrupt the cycle of dependence, and support smokers to become agents of change concerning their behavior. All participants received 4, 90- min structured weekly sessions during the smoking cessation phase of ST, and 6, 90-min ST maintenance sessions (at Weeks 6, 8, 10, 12, 24, and 48)	ST: 4, 90-min structured weekly sessions during the smoking cessation phase, and 6, 90-min maintenance sessions (at Weeks 6, 8, 10, 12, 24, and 48)	86 individuals (ST *n* = 42; MBRP *n* = 44) recruited by phone from a waiting list of an outpatient public health tobacco treatment service in a single city in Brazil. 50.34 (10.25) years old, 80.23% female, and 60.46% smoked over 10 cigarettes per day.	Baseline, 4-, 12-, and 24-week follow-ups.	The primary outcome was smoking abstinence (expired carbon monoxide, participants with a carbon monoxide of < 10 were considered abstinent). Secondary outcomes included levels of mindfulness (FFMQ-BR), craving (QSU), positive and negative affect (PANAS), and depression and anxiety (HAD Scale).	According to the CC analyses, abstinence rates tended to increase between Wk 4 and 12 in both groups (Wk 4: ST [*n* = 24, 88.9%], MBRP [*n* = 16, 66.7%]; Wk 12: ST [*n* = 17, 89.5%], MBRP [*n* = 12, 85.7%]; Wk 24: ST [*n* = 6, 66.7%], MBRP [*n* = 9, 81.8%]). The results of the analysis of secondary outcomes indicated reductions in craving (QSU Factor 2) in the MBRP group from Wk 4 to 12 (M = 17.583) and increases in levels of mindfulness (FFMQ-BR) (M = − 7.833). No other effects were observed for secondary outcomes.	Small sample size with large attrition rates through the 24-week follow-up in both groups (MPRB - 75%; ST - 78.57%).
Witkiewitz et al., 2019 [42]	MBRP + Active tDCS: 8, 120-min sessions with the first 30-min consisting of tDCS.	MBRP + sham tDCS: Standard MBRP + 8, 120-min sessions with the first 30-min consisting of tDCS.	134 adults interested in reducing their drinking. 59.5% male, 47.6% non- Hispanic white, 38.1% Hispanic, 52.3 (13.0) years old.	Baseline, post-treatment, and two-month follow-up	Primary outcomes were alcohol consumption (TLFB), craving (PACS), alcohol cue-reactivity (assessed using a visual cue task), and inhibitory control (assessed using a stop signal task).	No difference in attendance by condition. Significant reductions in drinks per drinking day over time. Significant dose effect for the number of groups attended. Significant effects of time and dose for the number of groups attended on percent heavy drinking days and cue reactivity. No effects of active versus sham tDCS on outcomes.	Low follow-up rate.
							Exclusion of
							individuals with history
							of severe alcohol
							withdrawal likely
							reduced variability in
							sample and likely
							reduced sample AUD
							severity. No biomarkers
							of drinking--just self-
							report.
Yaghubi et al., 2017 [43]	MBRP: 8, 120-min sessions.	TAU: Standard for opioid dependence (no substantive description of treatment provided)	70 Iranian adults receiving methadone for opioid dependence. 100% male, 20–45 years old.	Baseline, post-intervention, and 2-month follow-up.	Primary outcomes were impulsive behavior (BIS-11) and opioid (assessed using a biochemical morphine test).	MBRP predicted a significantly higher likelihood of abstinence relative to TAU at post-treatment and follow-up. MBRP also predicted a significant decrease in impulsivity among all subscales and total scores at post-treatment and follow-up.	The study may have limited generalizability because the sample was entirely male.
Zemestani et al., 2016 [44]	MBRP: 8, 120-min sessions.	TAU: 12-step, process oriented inpatient treatment. Topics included psycho-education, effects of substance use on interpersonal relationships, rational thinking skills, relapse prevention skills and related themes.	74 Iranian adults receiving inpatient treatment for substance use disorder. 79.7% male, 30.1 (9.4) years old.	Baseline, post- intervention, and 2-month follow-up.	Primary outcomes were depression and anxiety symptoms (BDI-II and the BAI) and craving (PACS).	Participants in the MBRP condition reported significantly lower post-intervention rates of depression, anxiety, and craving as compared to those in TAU. These results were consistent at follow-up.	Many patients did not meet criteria for a major depressive disorder
Zgierska et al., 2019 [45]	MBRP (tailored to AUD) + standard care: 8, 120-min sessions plus 6+ days per week formal (e.g., body scan for 30 min) and informal (mindfulness of activities) practice.	TAU: Individual and/or group outpatient therapy for alcohol use disorder. Primary modalities: 12-step facilitation, motivational enhancement therapy, relapse prevention, cognitive behavioral therapy. Encouraged to participate in mutual self-help meetings	112 adults receiving inpatient treatment for alcohol dependence. 56.2% male, 91% non-Hispanic White, 41 (12.2) years old	Baseline, post-treatment, and 18-week follow-up	Primary outcomes were: alcohol consumption (TLFB), alcohol-related consequences (DrInC), stress (PSS), and mindfulness (MAAS).	No significant differences between conditions at post-treatment and follow-up.	Small sample size and there was no blinding. Only one therapist provided all of the treatment.
Mindfulness Oriented Recovery Enhancement (MORE)							
Garland et al. 2016 and Garland et al. 2018 (erratum) [15, 46]	MORE: 10, 120-min sessions.	TAU: Groups at the therapeutic community (approximately 2 h/day). Consisted of psychoeducation, coping skills, etc. CBT: 10, 120-min group sessions. Addressed co-occurring SUD and PTSD via skills training (i.e., not exposure-based).	180 men with co-occurring psychiatric and SUD (MORE = 64, CBT = 64, TAU = 52) who were previously homeless, currently resided in a therapeutic community, and had histories of trauma. MORE: 37.7 (SD = 10.4) years old, 40% non-Hispanic white. CBT: 36.5 (SD = 11.2) years old, 44% non-Hispanic White, TAU: 38.7 (SD = 9.8) years old, 42% non-Hispanic white.	Baseline and post-treatment.	Primary outcomes were craving (PACS), PTSD symptoms (PCL), psychiatric distress (BSI). Secondary outcomes were mindfulness (FFMQ) and positive and negative affect (PANAS).	Craving decreased in all treatments, with greater decreases in MORE, as compared to CBT, but not as compared to TAU. MORE also produced greater decreases in PTSD symptoms than CBT and TAU. Anxiety and depression symptoms decreased across all groups, but there was not a significant treatment x time interaction effect for these outcomes. MORE produced greater increases in mindfulness, as compared to control conditions. MORE produced greater increases in positive affect, as compared to TAU, and decreases in negative affect, as compared to CBT. Changes in mindfulness were significantly negatively correlated with changes in craving and PTSD symptoms and changes in mindfulness mediated the effect of MORE (vs. control conditions) on decreases in craving and PTSD symptoms.	No post-treatment follow-up assessments. Limited generalizability (i.e., to women, individuals not residing in a therapeutic community, etc.)
Garland et al., 2017 [47]	MORE 8, 120-min sessions.	Support group (SG); 8, 120- min sessions. The topics were pertinent to chronic pain and long-term opioid use and included: the physical and emotional aspects of pain experience, ways of coping with chronic pain, ways of coping with negative emotions, the effect of life stress on pain, the stigma of opioid use, risks of long-term opioid use and opioid dependence, acceptance versus denial, and plans for the future.	115 prescription opioid users (MORE = 57 randomized; SG = 58) for pain relief on a daily or near-daily basis for at least 90 days, and self- reported chronic pain conditions including low-back pain, fibromyalgia, arthritis, cervical pain, or other pain conditions unrelated to cancer. 67.8% female, 48.4 (SD = 13.6) years, 65% non- Hispanic white.	Baseline, post- treatment, 3-month post-treatment follow-up	The primary outcome was opioid attentional bias (AB) as measured by the dot-probe task.	The pretreatment 200 ms opioid AB status significantly moderated the effect of the treatment condition on post-treatment opioid AB, indicating that the effects of treatment significantly differed by pretreatment opioid AB status. There were no significant effects of the treatment condition on post-treatment 2000 ms opioid AB found, nor did pretreatment opioid AB serve as a moderator of treatment effects. The 2000 ms opioid AB did not change from pretreatment to post-treatment, but the reductions in 200 ms opioid AB over the course of treatment predicted lower levels of opioid misuse at 3- month follow-up, suggesting that decreasing attentional fixation on opioid cues may reduce risk of future opioid misuse.	Participants were chronic pain patients that did not have to meet the criteria for OUD. Exploratory secondary analysis with relatively high attrition rates (*n* = 72; 63% of the randomly allocated sample, 81% of those who attended one or more sessions) completed the treatment.
Garland et al., 2017 [48]	MORE: 8, 120-min sessions.	Support group (SG): 8, 120- min sessions. The topics were pertinent to chronic pain and long-term opioid use and included: the physical and emotional aspects of pain experience, ways of coping with chronic pain, ways of coping with negative emotions, the effect of life stress on pain, the stigma of opioid use, risks of long-term opioid use and opioid dependence, acceptance versus denial, and plans for the future.	55 chronic pain patients. This subset was taken from a larger sample (115) during an RCT testing the effectiveness of the MORE intervention for individuals with chronic pain. MORE *n* = 26, SG *n* = 29, 21 men and 34 women, 48.9 (11.6) years old.	Participants completed EMA via a daily diary in which they rated their current pain level and affect rating each time they took their opioid dose with ratings accepted up to four times each day. Baseline and post-treatment assessment of opioid misuse.	The primary outcomes were pain intensity and affective state (collected via EMA) and aberrant medication-related behavior (COMM).	Aggregated over time, patients in the MORE group showed a 7% reduction in pain, compared to a 3% increase in pain among the SG; thus pain intensity improved over the 8 weeks of MORE relative to the SG (an estimated improvement of 0.7 points on a scale of 0–10). Over the 8 weeks of the study interventions, participants in MORE were 2.75 times more likely to be affectively regulated than participants in the SG. Mean scores on the COMM decreased during treatment by 7.06 in the MORE group and by 3.25 in SG.	The population was not exclusively comprised of opioid misusers (67.3% had COMM scores greater than or equal to 13). Specific response rate to the EMA was not reported. The inclusionary criteria required completion of at least one EMA measurement.
Garland et al., 2017 [49]	MORE: 8, 120-min sessions.	Support group (SG): 8, 120- min sessions. The topics were pertinent to chronic pain and long-term opioid use and included: the physical and emotional aspects of pain experience, ways of coping with chronic pain, ways of coping with negative emotions, the effect of life stress on pain, the stigma of opioid use, risks of long-term opioid use and opioid dependence, acceptance versus denial, and plans for the future.	51 chronic pain patients (MORE *n* = 20, SG *n* = 31). This subset was taken from a larger sample (115) during an RCT testing the effectiveness of the MORE intervention for individuals with chronic pain. 17 men and 34 women, mean age = 45.7 (13.7) years old.	Baseline and post-treatment.	The primary outcome was changes in relative responsiveness to natural reward and opioid cues from pre- to post-intervention. HR was used to measure cue responsiveness and high- frequency heart rate variability (HRV) to index parasympathetic regulation of hedonic responses, including attention to emotional information. Positive scores indicated increased natural reward cue-elicited HR relative to opioid cue-elicited HR. Negative scores indicated decreased cue-elicited HR to natural reward cues compared with opioid cue-elicited HR.	There was a significant group × time effect on HRV responsivity, indicating that compared to the SG, the MORE group experienced significantly greater increases in HRV responsivity during affective picture viewing. This would indicate that MORE may be effective at enhancing autonomic regulation of stress created by drug-related stimuli. MORE participants experienced greater reductions in cue-elicited HR. This effect was most prominent for drug cue-elicited HR. This finding may point to the downregulation of response to drug-related cues.	Small sample size. There were only 51 individuals (of the larger 115) who had complete sets of data for this exploratory hypothesis. Participants were chronic pain patients that did not necessarily meet the criteria for OUD.
Garland et al., 2019 [50]	MORE: 8, 120-min sessions held once a week.	Support Group (SG): 8, 120- min sessions held once a week. Included discussions on topics pertinent to chronic pain and long-term opioid use including: the lived experience of chronic pain, ways of coping with chronic pain; ways of coping with negative emotions, how stressful life events impact pain, the stigma of opioid use and dependence, adverse effects of opioids, acceptance versus denial, and plans for the future.	95 participants recruited from primary care and pain clinics in Salt Lake City, Utah. 56.8 (11.7) years old, 66% female, 89% non-Hispanic White, 122.87 (93.6) months of opioid use.	Baseline, post-intervention, and 3- month follow-up.	Primary outcomes were: positive affect (PANAS), meaning in life (MLQ), savoring (MSS), self- transcendence (NADA), pain severity (BPI), and opioid misuse risk (COMM).	There was a significant effect of intervention group on change in positive affect, meaning in life, savoring, and self-transcendence, indicating that MORE increased these attributes to a greater extent than did the SG. For the clinical variables, there was a significant effect of intervention group on residualized change in pain at post-treatment and change in opioid misuse risk by 3-month follow-up, such that MORE resulted in greater improvements in these variables than did the SG.	The participants were opioid-treated chronic pain patients and did not necessarily meet the criteria for OUD. Attrition rates were high (47.4%), but comparable to that of other opioid interventions.
Garland et al., 2019 [51]	MORE + MMT: 8, 120-min sessions.	TAU + MMT: 8, 120-min sessions. TAU consisted of individual and group therapy provided by participating in MMT treatment agencies. TAU therapeutic approaches included process-oriented, present-centered therapy, and cognitive-behavioral coping skills training but did not include formal mindfulness-based intervention.	30 individuals enrolled in an MMT program (MORE = 15; TAU = 15). 50.4 (8.44) years old, 50% female, 36.6% non- Hispanic White, 67% reported using heroin in the past 30 days, and had received a median of 7 months of MMT (range < 1 month to 35 years).	112 EMA collection points (2 assessments per day/ 56 days).	Primary outcomes were craving and urge to use opioids, pain intensity, and affective state. The secondary outcome was event contingent craving.	MORE participants reported greater decreases in several categories including: opioid wanting (44% decrease), opioid urge (50% decrease), pain unpleasantness (13% decrease), and stress (26% decrease). Event-contingent EMA showed MORE participants to report a greater number of cravings (*n* = 303) than participants in TAU (*n* = 87). Yet, those cravings were reported as being significantly less intense than those experienced by participants in TAU. Participants in MORE had 68% less severe opioid wanting and 56% weaker opioid urges than participants in TAU.	The daily response rate to random EMA probes over the two months of the intervention was 62%.
Garland et al., 2019 [52]	MORE: 8, 120-min sessions.	Active Support Group (SG): 8, 120-min. Client-centered group format that facilitated emotional expression and discussion of topics pertinent to chronic pain and opioid use/misuse.	135 individuals recruited from primary care and pain clinics in Salt Lake City, UT (Sample 1 = 40, Sample 2 = 31, Sample 3 = 64). 55.4 (11.1), 57.8 (11.3), 56.7 (10.9) years old respectively per sample. 57.5, 12.9, and 65.6% female. 90, 80.6, and 82.8% non-Hispanic White. Average duration of opioid use, 10.13 (7.17) years	Baseline and post-intervention.	Experiment 1 assessed the effects of treatment on LPP indices of opioid cue-reactivity relative to reactivity to neutral cues. Experiment 2 assessed the effects of treatment on the capacity to down-regulate the LPP response to opioid cues. Experiment 3 assessed the effects of treatment on the capacity to up-regulate the LPP response to natural reward cues. Experiment 4 evaluated affect ratings in response to natural reward cues collected from a sample of opioid-treated chronic pain patients participating in a stage 2 RCT of MORE.	For experiment 1, within-group comparisons indicated that SG participants’ post-treatment LPP activations remained higher in response to opioid cues compared to the neutral cues, suggesting an opioid cue-reactivity. Relative to those in the SG, participants who were treated with MORE exhibited significantly greater decreases in the LPP response to opioid cues during regulation (regulate < view) from pre-treatment to post-treatment. MORE participants showed significantly greater increases in LPP response to natural reward cues during regulation from pre-treatment to post-treatment. Relative to those in the SG, participants who were treated with MORE exhibited significantly greater positive affective responses to natural reward cues from pre-treatment to post-treatment.	The study was not a clinical trial powered to detect changes in treatment outcomes.
Mindful Awareness in Body-Oriented Therapy (MABT)							
Price et al. 2019 [23]	MABT: 8, 90-min sessions.	TAU: Group sessions 2- 3x/week for 10–14 weeks, individual counseling at least 1x/month. Included education about alcohol and drug use and participation in self-help groups (e.g., 12-step) for SUD. Women’s health education (WHE): 8, 90-min group sessions. Included topics such as understanding the female body, reproductive health, cardiovascular health, and sexually transmitted diseases.	Women (MABT = 93 initially, 74 included in analyses; WHE = 56 initially, 46 included in analyses; TAU = 68 initially, 67 included in analyses) recruited from outpatient SUD treatment programs who were currently enrolled in intensive outpatient treatment. Median age of 35 years, 75% non-Hispanic white.	Baseline and 3-month post-baseline follow-up	Substance use outcomes were % of days abstinent and a binary indicator of relapse (TLFB, urine drug screen, electronic health records) and craving (PACS). Mindfulness outcomes were interoceptive awareness (MAIA) and mindfulness (FIM). Psychiatric distress outcomes were DERS, psychophysiological response to a stressful video, rumination, and body awareness (Respiratory Sinus Arrhythmia; RSA), BDI-II, and PSS-SR. Also assessed patient satisfaction.	Participants who received MABT or WHE, compared with TAU, showed significantly greater improvement in the proportion of days abstinent; however, there were no significant differences across groups in relapse or craving. MABT produced significantly greater improvements in mindfulness, as compared to the control conditions, in analyses including individuals who attended 6+ groups, but not in intent to treat (ITT) analyses. MABT was associated with greater improvements in 6/8 MAIA subscales, as compared to the control conditions. MABT was associated with improvements in DERS scores and RSA during the film reactivity task, the body awareness reactivity task, and (in analyses of those who completed 6+ groups) resting RSA, as compared to the control conditions. As compared to control conditions, MABT was associated with greater improvements in BDI-II scores among those who completed 6+ groups, but was not associated with improvements in BDI-II or PSS- SR scores in ITT analyses. 72% of MABT and 63% of WHE participants endorsed highly positive treatment satisfaction ratings of “very much” or “extremely.”	Only examined short- term follow-up. Findings might not generalize to men.
Price et al. 2019 [53]	MABT: 8, 90-min sessions.	TAU: Group sessions 2-3x/week for 10–14 weeks, individual counseling at least 1x/month. Included education about alcohol and drug use and participation in self-help groups (e.g., 12-step) for SUD. Women’s health education (WHE): 8, 90-min group sessions.	Reported above.	Baseline, 3-, 6-,and 12-month follow-up.	The primary outcome was days abstinent from substance use (TLFB). Secondary outcomes included measures of emotion regulation (DERS), craving (PACS), psychological distress (PSS-SR and BDI-II), mindfulness (FMI), and interoceptive awareness (MAIA), respiratory sinus arrhythmia (Tonic RSA).	Primary Outcome: The ID sample showed a significant difference between groups. MABT & WHE PDA > TAU at 6 months, MABT > TAU at 12 months. MABT improvements maintained the increased level while WHE & TAU decreased over time. No differences between MABT and WHE at any time point. Secondary Outcomes: Self-report emotion dysregulation had no significant difference at any time point in ID and ITT analysis. Craving: overall ITT & ID showed significant improvements at 6- and 12-months. Psychological distress. No longitudinal difference in mindfulness. Overall significant difference in ID sample with increases in mindfulness skills at 3- and 6-months. Interoceptive awareness. Overall, a significant difference in ITT and ID samples at 3- and 6-months.	Low generalizability due to the low SES of participants, and the fact that all members were women. Study is re-reporting some results with an added time point.
Mindfulness-Based Addiction Treatment (MBAT)							
Spears et al., 2017 [26]	MBAT: 8, 120-min group sessions.	CBT (Manualized): 8, 120- min group session treatment for smoking cessation. Quit day scheduled for Session 5. UC: 4, 5–10 min counseling sessions. Intended to be equivalent to typical support from a health care provider. Content emphasized problem solving and coping skills training. Quit day scheduled for Session 3.	412 adults who were current smokers (average 5+ cigarettes per day for the past year), motivated to quit within 30 days. 54.9% female, 48.2% African-American, 41.5% non-Latino white. Mean age not reported.	Baseline, treatment weeks 3–8, 26- week follow-up	Smoking cessation outcomes were: nicotine dependence (HSI), smoking dependence and smoking withdrawal ((WISDM) and (WSWS)), smoking avoidance self-efficacy (SES), and attentional bias toward cigarettes(SBQ). Behavioral outcomes were: affect (PANAS), Depression symptoms (CES D), Stress (PSS), and expectations (AIPQ).	Participants in the MBAT condition perceived greater volitional control over smoking and lower volatility of anger than CBT and UC. No other significant differences nor significant indirect effects of MBAT vs CBT. Participants in the MBAT condition reported lower anxiety, attentional bias, concentration difficulties, craving, smoking dependence motives and higher self-efficacy for negative affect relative to UC.	There was a heavy reliance on repeated administration of self- report questionnaires with significant time lapses between assessments.
Vidrine et al., 2016 [25]	MBAT: 8, 120-min group sessions.	CBT (Manualized): 8, 120-min group session treatment for smoking cessation. Quit day scheduled for Session 5. UC: 4, 5–10 min counseling sessions. Intended to be equivalent to typical support from a health care provider. Content emphasized problem solving and coping skills training. Quit day scheduled for Session 3.	412 adults, current smokers (average 5+ cigarettes per day for the past year), motivated to quit within 30 days. 54.9% female, 48.2% African-American, 41.5% non-Latino white. Mean age not reported.	Baseline, post-treatment, 26-week follow-up	Nicotine dependence (HSI), self-report average days using mindfulness techniques, and smoking abstinence was biochemically confirmed.	MBAT participants reported the highest percentage of 7-day point prevalence abstinence, but not a significant difference. Among participants classified as smoking at final tx sessions, recovery of abstinence was examined. MBAT condition had significantly higher recovery than CBT (Cohen’s d = .88) and UC (Cohen’s d = .79)	The same therapists delivered all treatments. No assessment of fidelity
Mindfulness Training for Smoking Cessation (MTS)							
Kober et al. 2017 [16]	MTS: 8, 90-min sessions over 4 weeks.	Freedom From Smoking (FFS): 8, 90-min sessions over 4 weeks. Topics included cognitive strategies for coping with cravings and stress/negative emotions, behavior modification, and relapse prevention.	23 adults (MT = 11, FFS = 12) who smoked > 10 cigarettes/day, had fewer than 3 months of abstinence in the previous year, and were interested in smoking cessation. All participants were part of a larger trial comparing MT and FFS and participants included in the present analysis represent a subset who completed a neuroimaging task. 69.6% male, 48.3 (7.0) years old, 60.9% non-Hispanic white.	Baseline, post-treatment, and 3-month post-treatment follow-up.	Smoking outcomes were reductions in average cigarettes/day from pre- to post-treatment (TLFB). Also examined fMRI and self-reported stress reactivity and craving to stress/negative scrips and neutral/relaxing scripts.	Those in both conditions reduced smoking (cigarettes/day), but the MT group showed greater reductions during treatment and at the 3-month post-treatment follow-up. The MT and FFS groups did not differ in their self-reported stress or craving to stressful/negative scripts at the post-treatment fMRI session and there were no significant group differences in brain activity during neutral scenarios. Participants in the FFS group (vs. MT) exhibited increased neural reactivity in several brain regions during the stressful scripts. The MT group did not demonstrate greater neural reactivity in any region. Lower reactivity in several brain regions during stressful scripts in the MT condition (vs. the FFS condition) was associated with a greater reduction in smoking after treatment and at 3-month follow-up.	Small sample size. Only completed fMRI post-treatment, and therefore could not assess reductions in neural stress reactivity.
Moment-by-Moment in Women’s Recovery (MMWR)							
Black et al., 2019 [17]	Residential SUD treatment program + MMWR: 12, 80- min sessions delivered twice weekly. MMWR was delivered across 6 weeks (as opposed to the standard 12) to better fit the residential site clinical services structure. The residential program included: mental health and SUD diagnosis and treatment (individual and group), trauma education and support, vocational training, nutritional education and support, health and wellness activities, and 12-step meetings, among other services.	Neurobiology of addiction (NA): 12, 80-min group sessions delivered twice weekly. Topics included: definition of addiction and why it is a brain disease; brain structures and functions and those related to addiction; effects of various types of substances on the brain; rewarding effects of substances and how these effects lead to addiction; definitions and brain functions related to craving and withdrawal; and the roles of treatment in recovery.	200 female patients (MMWR- 100, NA-100) at a residential SUD treatment facility. 32.5 (9.1) years old, 21.0% non- Hispanic White, 46.5% had less than a high school education, 62% had been incarcerated in the 8 months prior to residential treatment entry, and 76% were amphetamine/methamphetamine- mine users	Baseline and post-intervention assessment of the self-report measures. 150-day cap (beginning on the first day of the intervention) for discharge status (completer, non- completer with satisfactory progress, non- completer without satisfactory progress, in- residence).	The primary outcome was discharge status and days until discharge. Secondary outcomes were self-report measure scores of study intervention mechanisms of action: mindfulness (FFMQ-SF), perceived stress (PSS-10), distress tolerance and emotion regulation (DERS), distress (DASS-21), affect (PANAS), and drug and alcohol craving (PACS).	The hazard ratio for retention was of medium-to-large effect size, suggesting the clinical relevance of adding MMWR to an all-women’s, ethno-racially diverse, SUD residential treatment center. The length of stay in residential treatment from study intervention start date to analytic endpoint (150 days) was 94.4 days. There were 74 treatment completers (43/100 in MMWR, 31/100 in the NA group), 42 women still in residence (15/100 in MMWR, 27/100 in the NA group). There were 84 treatment non-completers, but satisfactory progress was made by 16/42 women in the MMWR group and 10/42 women in the NA group. For the ITT, risk of non-completion without improvement was lower in MMWR as compared to the NA group after the study intervention. At post-intervention, all eight secondary outcome measurements had improved for both groups.	It is possible that the control curriculum was equally beneficial to some self-reported measures of therapeutic change, which was evident for significantly improved scores in both groups for the FFMQ, DTS, DERS, and PACS.

### Mindfulness-based relapse prevention (MBRP)

#### Overview of protocol

MBRP is a manualized, structured protocol that integrates formal meditation practices with the cognitive behavioral approach of relapse prevention treatment. The core goals of MBRP are to: 1) cultivate awareness of internal (e.g., emotions, thoughts) and external (e.g., environmental) substance use cues to create opportunities to address triggers rather than instinctively using substances; and 2) practice (both via imagination and in vivo exposure) remaining with unpleasant affective, cognitive, or physical experiences without automatically seeking to escape or avoid the situation [13, 54]. MBRP courses are typically in a group format and consist of eight, 120-min sessions. Each session begins with a guided mindfulness practice and is followed by “inquiry,” a discussion (similar to Socratic questioning) about the client’s present moment experience of the practice. The first session uses experiential exercises focused on bodily sensations (e.g., taste and smell) designed to introduce the rationale for mindfulness by examining the role that “autopilot” plays in daily life and contrasting it with mindful awareness of experiences. The second and third sessions shift to other aspects of current experience such as sight and sound. Clients are encouraged to notice triggers and urges for substance use and to practice exercises throughout the day to encourage exiting autopilot and increasing mindfulness. Sessions four through six focus on mindfulness of thoughts and emotions. For example, clients begin to bring awareness to patterns of behavior and antecedents to relapse and are taught to notice thoughts, sensations, or emotions that might arise while remaining focused on the present moment experience. Clients are instructed to remain mindful of the present moment despite the unpleasant sensation as a method of allowing time and space to make a less impulsive, skillful decision. In sessions seven and eight, clients discuss generalizing learned practices and facilitating an environment that can support continued practice of mindfulness and changes made to substance use.

MBRP is one of, if not the most, researched MBI for SUD. Bowen and colleagues [55] conducted an initial pilot feasibility and efficacy trial of MBRP. They found MBRP to be feasible to implement and found initial support for efficacy as measured by significantly lower substance use rate, decreases in craving, and increases in acceptance and acting with awareness as compared to TAU. Other large scale RCTs and meta-analyses have shown the efficacy of MBRP with a range of samples (e.g., [56, 57]), treatment delivery modalities (e.g., [13, 56, 57]), and treatment targets [14, 56, 58]. Recent research includes RCTs designed to replicate or expand earlier findings (i.e., to new populations, examining adjunctive interventions) and secondary data analyses that seek to identify potential mechanisms of change and treatment moderators.

#### Literature review

##### Expansion of MBRP findings: specific populations, modified protocols, and adjunctive interventions

Several recent studies have aimed to expand previous findings on MBRP to specific populations with SUD. For example, Glasner-Edwards and colleagues compared MBRP to health education (control condition) among stimulant dependent adults (DSM-IV criteria) receiving a 12-week, 24 session, contingency management intervention (*n* = 63) [35]. MBRP sessions were shortened to 75 min to increase engagement with this population. There were no significant differences between MBRP and control on negative urinalysis rate or Addiction Severity Index score at post-treatment and one-month follow-up. However, MBRP was associated with lower odds of stimulant use among individuals with depressive and anxiety disorders. Participants in the MBRP condition reported greater reductions in depressive symptoms (Cohen’s *d* = 0.58) and psychiatric severity (Cohen’s *d* = 0.61) over time relative to control.

Modifications of MBRP for specific substances have also recently been evaluated. In an RCT comparing the efficacy of MBRP for alcohol use disorder (adapted to focus on alcohol-specific content) relative to TAU among adults in outpatient substance use treatment, Zgierska and colleagues did not find significant post-treatment differences on any of the constructs of interest (alcohol consumption, drinking-related consequences, perceived stress, or mindfulness) [45]. Weiss de Souza and colleagues compared the effects of MBRP in conjunction with the Brazilian Ministry of Health’s tobacco cessation standard treatment (ST) protocol to the stand-alone ST protocol [41]. The intention to treat (ITT) analysis showed that at week 24 abstinence was 14.3% for ST, but 20.1% for MBRP, indicating that continued smoking rates were 37% lower in the MBRP group. The secondary outcomes showed that individuals in the MBRP group had reductions in craving based on the Questionnaire of Smoking Urges (QSU) from week 4 to week 12 (Mean = 17.58) and increases in levels of mindfulness based on the Five Facet Mindfulness Questionnaire-Brazil (FFMQ-BR) (Mean = − 7.83).

Three recent studies have also demonstrated initial efficacy of MBRP among additional international samples, including those with opioid use disorder and co-occurring depression. Yaghubi and colleagues compared the efficacy of MBRP relative to TAU among male, opioid-dependent patients referred for methadone maintenance treatment in Iran [43]. MBRP participants experienced higher rates of abstinence at post-treatment and at two-month follow-up relative to TAU participants. Impulsivity scores among MBRP participants decreased significantly over time relative to TAU. In addition, an eight-week intervention comparing MBRP to treatment as usual for Iranian males in methadone maintenance treatment (*n* = 60) was conducted by Abed & Shahidi [30]. Participants in the MBRP condition reported significant decreases in craving and desire to use. Lower levels of positive urinalysis tests were reported among MBRP participants over the three-month follow-up; however, significance testing was not conducted. Zemestani & Ottaviani compared the efficacy of MBRP relative to TAU among substance dependent (DSM-IV criteria), moderately depressed Iranian adults receiving outpatient treatment for substance dependence [44]. MBRP participants reported decreased depression, anxiety, and craving relative to TAU participants.

Recent trials have also focused on adapting MBRP to improve implementation and efficacy. In an effort to make MBRP implementation more practical, Shorey and colleagues compared blended MBRP and acceptance and commitment therapy (ACT) to TAU among adult, residential substance use patients [40]. The groups were adapted to be an open, rolling format, and multiple meetings were held per week so that participants could complete all eight sessions during their 28–30 day stay. The study did not find significant post-treatment differences between conditions on primary outcomes (craving, mindfulness, and psychological flexibility).

Furthermore, the efficacy of MBRP with active and sham transcranial direct current stimulation (tDCS) was compared among individuals interested in decreasing their alcohol use [42]. Witkiewitz and colleagues’ results demonstrated a decrease in drinks per drinking day over time among the sample as a whole and a significant dose effect for the number of groups attended. However, there were no effects of active versus sham tDCS on primary (drinks per drinking day) or secondary outcomes (percent heavy drinking days and alcohol cue reactivity). This suggests no additive effects of tDCS in enhancing MBRP among individuals seeking outpatient treatment for drinking. A secondary analysis of this trial was conducted to examine the impact of tDCS on hypersensitivity to alcohol cues (as measured by craving, negativity toward cues, and late positive potential) [31]. Brown and colleagues found that self-reported craving and late positive potential (LPP; an event-related brain potential that is thought to measure motivational and/or emotional salience) decreased when exposed to alcohol cues across all conditions over time. The magnitude of the reduction was associated with the number of MBRP sessions attended, such that participants attending a greater number of sessions reported decreased craving when exposed to alcohol cues.

##### Moderators of treatment outcome

In an attempt to extend previous findings on MRBP efficacy, recent secondary data analyses of large-scale trials have aimed to identify subgroups who may benefit the most from MBRP. A secondary data analysis of two separate MBRP RCTs was conducted to examine potential moderators of efficacy [38]. In one sample [59], participants with high SUD severity and high levels of anxiety and depression that received MBRP reported 96% fewer heavy drinking days and 94% fewer drug use days at 12-month follow-up relative to relapse prevention (RP) therapy and TAU. Participants with high SUD severity and low levels of anxiety and depression that received MBRP reported 96% fewer heavy drinking days at 12-month follow-up relative to relapse prevention and treatment as usual. In another sample [55], participants with high SUD severity and high anxiety/depression or high SUD severity and low anxiety/depression that received MBRP reported 99% fewer alcohol and other drug use days than TAU participants. Roos and colleagues’ results suggest that MBRP may be most effective for individuals with high SUD severity *or* for individuals with high SUD severity and high levels of co-occurring anxiety and depression symptoms.

Two recent analyses have also examined demographic factors as moderators of treatment outcome. Roos and colleagues conducted a secondary data analysis of an MBRP RCT [59] to examine individual gender and group gender composition as a moderator of efficacy [39]. Participants in the MBRP condition reported 84% fewer drug use days at 12-month follow-up relative to relapse prevention participants. There were no significant main effects of individual gender or group gender composition. There was a significant group by gender interaction such that individuals in MBRP groups with one-third or more women reported 100% fewer drug use days at 12-month follow-up relative to relapse prevention participants.

With respect to racial/ethnic identity, Greenfield and colleagues conducted a secondary analysis of the initial MBRP pilot trial [55] examining the role of individual identity and racial/ethnic group composition among individuals receiving an MBRP intervention after discharge from an intensive outpatient unit or inpatient treatment [37]. White participants in the MBRP condition had lower heavy drinking days relative to relapse prevention (control condition), but no treatment difference in drug use days. Minority participants did not have significant treatment differences in heavy drinking days but did have lower drug use days in the MBRP condition than control. Racial/ethnic group composition was a significant moderator with participants in groups that were more than half white exhibiting lower heavy drinking days in MBRP than control, whereas participants in groups that were more than half minority showed no treatment difference in heavy drinking days. The group composition interaction was non-significant for drug use days.

##### Mechanisms of behavior change

Several recent studies have examined factors that mediate the effect of MBRP on substance use outcomes, including reductions in stress, impulsivity, craving, and stress reactivity. Davis and colleagues conducted a trial among low-socioeconomic status young adults in a residential substance use treatment facility (*n* = 79) [33]. Participants were randomly assigned to receive either eight sessions of MBRP or eight social support group sessions (e.g., Alcoholics Anonymous) in addition to typical treatment during their residential stay. At post-treatment, young adults receiving MBRP reported lower levels of substance use (Cohen’s *d* = − 0.58), craving (Cohen’s *d* = − 0.58), and stress (Cohen’s *d* = − 0.77) relative to TAU participants. Stress reduction during treatment partially mediated observed outcome differences between MBRP and TAU for substance use, suggesting that the effects of MBRP on long-term substance use may be at least partially explained by reductions in stress.

The efficacy of MBRP at decreasing impulsivity was compared to TAU and 12-step/self-help among young adults in residential SUD treatment [34]. Davis and colleagues found that MBRP participants reported significantly lower negative urgency throughout treatment (Cohen’s *d* = − 0.26) and maintained the improvement at follow-ups (Cohen’s *d* = − 0.51 and − 0.21) relative to TAU. All participants reported significantly lower positive urgency during treatment and MBRP participants maintained these gains at follow-up (Cohen’s *d* range from − 0.37 to − 0.10). MBRP participants reported significantly lower lack of premeditation (Cohen’s *d* ranged from − 0.66 to − 0.43) and lack of perseverance (Cohen’s *d* ranged from − 0.43 to − 0.16) during treatment but did not maintain this change at follow-up. There were no differences between conditions on sensation seeking. Additionally, positive and negative urgency mediated the relationship between treatment and substance use, such that participants in the MBRP condition had greater reductions in urgency and were then less likely to use the substance over time. The results suggest that MBRP is an effective intervention for young adults with SUD in residential treatment and that positive and negative urgency may be a mechanism of change.

A secondary data analysis of two separate MBRP RCTs was conducted to examine if the finding of mindfulness mediating the effect of MBRP on craving replicates in a new sample of individuals who completed the same measures [36]. In one sample [55](Study 1), the effect of MBRP on psychological flexibility, craving, and mindfulness was small to medium (Cohen’s d ranged from 0.08 to 0.48) and much smaller in the other sample ([59] Study 2; Cohen’s d ranged from 0.03 to 0.21). In Study 1, participants had higher scores on these mindfulness measures at post-treatment relative to TAU, and the post-treatment latent mindfulness factor significantly mediated the associations between MBRP and craving. In Study 2, participants in the TAU condition had higher scores on the three mindfulness measures at post-treatment relative to MBRP, and there was no mediating effect of mindfulness.

Carroll & Lustyk used a cognitive stressor to compare its impact on reactivity (measured by tonic and phasic heart rate variability, blood pressure, self-reported anxiety, self-reported craving, and heart rate) among adults who had completed 8 weeks of MBRP (*n* = 12), RP (n = 12), or TAU (weekly process-oriented groups regularly provided by the community treatment agency; *n* = 10) [32]. MBRP was related to higher levels of tonic and phasic heart rate variability, lower self-reported anxiety, and lower heart rate reactivity (than TAU only). There were no significant treatment effects on blood pressure or craving. Although formal mediation was not evaluated, the results suggest that participants who completed MBRP are still engaging with stress, but are cued to respond to stress in an adaptive, skillful manner. These processes might help to decrease substance use by increasing the use of adaptive coping skills.

### Mindfulness oriented recovery enhancement (MORE)

#### Overview of protocol

Mindfulness Oriented Recovery Enhancement (MORE) integrates elements of mindfulness training, cognitive restructuring, and positive psychology to address the factors theorized to maintain SUD [22]. More specifically, the three foundational elements of MORE are mindfulness, reappraisal, and savoring [22]. *Mindfulness* is described as a way to identify triggers and break the habit of automatic engagement in substance use in response to those triggers. *Reappraisal* is the process by which an individual reassesses a stressful event to be something more positive and constructive, as opposed to negative and destructive. *Savoring* is the act of focusing one’s attention on the positive, pleasing, and growth-inducing elements of an event. These foundational elements of MORE are the guiding principles that influence the ten-session protocol.

The standard protocol includes 10, 120-min group sessions that guide participants through the three foundational elements. The first three sessions of the MORE protocol are focused on laying the groundwork for the course by introducing the three foundational elements of MORE. In addition to introducing the practice of mindfulness, the first session presents the idea of automaticity, or “autopilot,” and the relationship between automaticity and engaging in substance use [22]. During sessions one through three, clients are asked to engage in a variety of experiential practices that range from focused attention of the breath, reframing an ordinary encounter on the street, to mindfully savoring an object in one’s immediate field of vision.

Sessions four through seven are centered around the Buddhist notion of impermanence, and the themes build upon the foundation of mindfulness. Session four and five are concentrated on the role that craving plays in substance use. Clients are asked to see the fleeting nature of any feeling, craving included, and to allow those temporary feelings to move through one’s body and mind without engaging with them or trying to change them. Clients experience the nature of craving when interacting with an object that is perceived as a treat (a piece of chocolate), then observe those same feelings of craving during a stressful situation [22]. Session six introduces the role that aversion plays in the recovery process, and how urges can be intensified when one tries to push away thoughts about drugs or alcohol. Session seven builds upon the nature of impermanence by highlighting the fleeting nature of our physical bodies and stresses the deleterious effects that toxic substances and health-risk behaviors have on our fragile existence. The primary exercise used during this session is the *Impermanent Body Exercise*. This practice asks the participants to bring an awareness to the facts that we will all get sick, we will all age, and we will all eventually die.

The final three sessions focus on how to interact with the environment following treatment and navigate back to the three core components of MORE. Session eight revisits the concept of autopilot and focuses on how one could react differently to a situation that would have previously driven engagement in substance use. The awareness of triggers and the use of mindfulness to break the chain of automatic action is the key focus of this session. Session nine returns to savoring, and highlights the beautiful nature of the world around us and how all things are connected in some way. Clients are encouraged to see the interdependent nature of life and simply to enjoy that beautiful connection we all share. The primary practice used to demonstrate this interconnection is the *Tasting Interdependence* exercise. In this practice, clients are given a raisin and are asked to follow the span of that raisin’s life from a grape seed planted into the soil up until the current moment as a dried piece of fruit. Session ten closes the course by recapping what has been learned, and how that new knowledge can be integrated into daily life.

MORE was originally developed and tested during an NIH-funded Stage 1 RCT for alcohol dependence. That study demonstrated that MORE could significantly improve physiological recovery from stress and drinking-related triggers [22]. Since that initial pilot trial of the MORE protocol, the program has been adapted and modified to fit the needs of various addictive behaviors such as opioid use disorder (OUD), internet gaming disorder, and chronic pain with prescription misuse.

#### Literature review

One of the primary processes that MORE targets is the urge to engage in substance use (i.e., craving). A large-scale RCT compared MORE to Cognitive Behavioral Therapy (CBT) and TAU in 180 male patients with co-occurring psychiatric and substance use disorders [15, 46]. Garland and colleagues found reductions in craving in all three conditions, with greater reductions in MORE than CBT, but not TAU. In addition to a reduction in craving, MORE was associated with greater decreases in post-traumatic stress symptoms versus both CBT and TAU.

Several recent trials have leveraged ecological momentary assessment (EMA) to explore the impact of MORE on craving, pain, and affective states. In one stage 1 RCT, EMA was utilized to examine the effects of MORE versus TAU on opioid craving, pain, and positive affect on individuals enrolled in methadone maintenance therapy (MMT) [51]. Compared to TAU, participants in MORE reported significantly greater decreases in opioid wanting (44% decrease) and opioid urge (50% decrease). EMA data also showed that participants in MORE reported a greater number of cravings (*n* = 303) than participants in TAU (*n* = 87), but MORE participants reported those cravings as being significantly less intense than those experienced by individuals in TAU. Participants in MORE also reported having 129% greater self-control over cravings than participants in TAU. Garland and colleagues also collected EMA data from a subset of individuals (*n* = 55) from a larger RCT to test the efficacy of MORE versus a support group (SG) at reducing perceived pain and improving affective state [48]. Participants were chronic pain patients with daily, to near daily, use of prescription opioids. Through the 8-weeks of the intervention, participants in the MORE group showed a 7% reduction in pain, compared to a 3% increase among the SG; thus pain intensity improved over the 8 weeks of MORE relative to the SG. Over the same duration, participants in MORE were 2.75 times more likely to be emotionally regulated than participants in the SG. Mean opioid misuse scores decreased during treatment by 7.06 in the MORE group and by 3.25 in SG.

Another mechanism that the MORE program may affect is cue reactivity. One study evaluated the impact of MORE versus a SG on the late positive potential (LPP) index of opioid cue-reactivity relative to reactivity to neutral cues in 135 individuals with chronic opioid use [52]. The LPP was used to measure prolonged attention to the opioid cues. Garland and colleagues found that for individuals in the SG, post-treatment LPP remained significantly higher in response to opioid cues compared to the neutral cues. Participants in MORE exhibited significantly greater decreases in the LPP response to opioid cues and also exhibited greater increases in the LPP response to natural reward cues from pre- to post-treatment. In another study, Garland and colleagues tested the relative responsiveness to natural reward and opioid cues in 51 individuals who used prescription opioids [49]. There was a significant group × time effect on heart rate variability (HRV) responsivity, indicating that compared to the SG, the MORE group experienced significantly greater increases in HRV responsivity in response to drug elicited cues.

Garland and colleagues also conducted a secondary data analysis to compare the effects of MORE on opioid attentional bias (AB) compared with a SG in 115 individuals who used prescription opioids [47]. The pre-treatment opioid AB significantly moderated the effect of the treatment condition on post-treatment opioid AB, indicating that the effects of treatment significantly differed by pre-treatment opioid AB. The post-treatment opioid AB score for MORE and SG participants (with pre-treatment opioid AB as a covariate) revealed a statistically significant effect of the treatment condition, with MORE participants showing significantly lower levels of opioid AB at post-treatment than SG participants. The reductions in opioid AB over the course of treatment predicted lower levels of opioid misuse at 3-month follow-up. All of these findings suggest that MORE may sever associations between conditioned drug cues and their reward value via bottom-up mechanisms and that decreasing attentional fixation on opioid cues may reduce the risk of future opioid misuse.

In an analysis of proximal outcome data collected from a larger RCT, Garland and colleagues looked at the effectiveness of MORE versus a SG on pain severity, opioid misuse, positive affect, meaning in life, and self-transcendence in 95 opioid treated chronic pain patients [50]. They found that there was a significant effect of intervention group on change in positive affect, meaning in life, savoring, and self-transcendence, indicating that MORE increased these attributes more so than the SG. For the clinical variables, there was a significant effect of intervention group on change in pain at post-treatment and change in opioid misuse risk by 3-month follow-up, such that MORE resulted in greater improvements in these variables than did the SG.

### Mindful awareness in body-oriented therapy (MABT)

#### Overview of protocol

Mindful Awareness in Body-Oriented Therapy (MABT) is a manualized, mindfulness-based approach that is designed to teach interoceptive skills for self-care [23]. Interoception is the ability to process signals that originate in the body and is broadly described as the overall sensations, or state, of the body [60]. MABT is different from most mindfulness-based practices in that it specifically focuses on teaching the client mind-body skills that are designed to help with a dysregulated awareness of sensory information. There appears to be a link between a deficient interpretation of sensory information and poor emotion regulation [24]. The intervention involves eight, 90-min group sessions over eight consecutive weeks. To meet the primary aim of MABT, the course is broken down into three specific stages. Stage 1 (sessions 1 and 2) is geared toward identifying bodily sensations (*identification*). Often clients are unaware that physical sensations can be identified, and so, the first two sessions are aimed at teaching body literacy through a response to expected sensations (i.e. the client grasping her arm and feeling the pressure created) [24].

Stage 2 of the MABT program (sessions 3 and 4) is directed at learning and developing strategies for interoceptive awareness (*access*), which is a more advanced aspect of body literacy that adds upon the skills learned in the first stage [23]. This stage focuses on sensations that are still quite overt and apparent, such as the sensation of breathing in and out, but clients are beginning to refine their bodily awareness. Clients progress from sensing continuous movements in the body to perceiving specific areas of the body (chest, foot, etc.) to develop the skills of interoception.

The final stage of MABT (sessions 5 through 8) is aimed at developing the ability to maintain/sustain interoceptive awareness throughout one’s daily life (*appraisal*) [23]. The ability to sustain this awareness of bodily sensations allows the client to detach oneself from the uncomfortable physical sensations that may be occurring in a situation, and allow themselves to reframe the experience more positively. The ability to investigate and determine the origin of the physical sensations that are occurring during a particular event, or emotional state of being, allow the client to take charge of their experience and perform the necessary actions for self-care that are more productive than engaging in substance use.

#### Literature review

In a large-scale RCT of 217 women enrolled in three separate outpatient treatment facilities, researchers determined if MABT resulted in improved interoceptive awareness and mindfulness skills (primary outcomes), emotion regulation (self-report and psychophysiology), symptomatic distress (e.g., depression and trauma-related symptoms), and substance use and craving compared with women’s health education (WHE), and TAU alone [23]. Price and colleagues found that MABT produced significantly greater improvements in mindfulness from pre- to post-intervention among participants who completed six or more sessions, as compared to the other groups. However, this effect was not significant in ITT analyses, including all participants with any available data. MABT also produced greater improvements in most factors of interoceptive awareness, including noticing, attention regulation, emotional awareness, self-regulation, body listening, and trust, as compared to the other two groups. However, MABT did not result in greater improvements in depression or trauma symptoms, both secondary outcomes, compared to the other two treatment groups (symptoms improved in all groups). Participants in both MABT and WHE conditions, compared with TAU, showed significantly greater improvement in the proportion of days abstinent; however, there were no significant differences across groups in relapse or craving.

A longitudinal follow-up of the previously mentioned RCT examined the number of days abstinent, overall psychological well-being, interoceptive awareness, and mindfulness skills across the three conditions through 12-month follow-up assessments among 187 of the original 217 participants [53]. Looking at the primary outcome of days abstinent from substance use among those who completed at least six sessions of treatment, the MABT and WHE groups showed a greater number of days abstinent compared to the TAU group at the 6-month and 12-month follow-up. The improvements in the number of days abstinent among MABT participants were maintained from 3 to 12 months, whereas TAU and WHE showed continuous declines in abstinent days, particularly at 12 months. There were no group differences observed for MABT vs. WHE in the percent of days abstinent or relapse at any assessment time-point.

### Mindfulness-based addiction treatment (MBAT)

#### Overview of protocol

Mindfulness-Based Addiction Treatment (MBAT) is a protocol that closely follows the procedures and rationale of Mindfulness-Based Cognitive Therapy (MBCT). Wetter and colleagues took most of the material from the MBCT handbook, but they removed the depression focused content and replaced it with information regarding substance use (e.g. smoking cessation material, alcohol abstinence, etc.) [25]. Three primary aims lead the eight, 120-min group sessions, including: (1) becoming aware of thoughts, emotions, and physical sensations from moment to moment, (2) creating a different way of relating to those thoughts, emotions, and physical sensations, and (3) using ones new-found awareness to separate oneself from those various occurrences that are arising [25]. Like the other mindfulness-based protocols reviewed, the primary aim of MBAT is to awaken clients to the automatic nature of substance use by teaching them the skills of noticing present moment feelings and sensations and detaching themselves from urges to engage in substance use.

The MBAT course is broken down into two primary sections. Sessions 1–4 are geared toward teaching clients how to direct and focus their attention [26]. This is done using a crawl-walk-run framework that has participants learn to focus their attention on something that is constantly occurring regardless of whether attention is paid to it or not, such as the sensation of breathing. Sessions 5–8 are aimed at using the skills of focused attention to help navigate through difficult situations that are related to substance use. Clients are taught specific techniques, primarily one titled “breathing space,” to find some room between stimulus and response [25]. For example, the group may be lead through a visualization exercise in which clients picture a situation where engaging in substance use would have been automatic. Clients are asked to pause the scenario just before visualizing engagement in substance use and are directed to instead observe the breath. This pause allows clients to detach from their feelings of craving and gives them the necessary space to investigate what is arising in them physically and emotionally.

#### Literature review

In a large-scale RCT, Vidrine and her research team wanted to test the effects of the MBAT protocol on smoking cessation compared with CBT and a usual care (UC) condition comprised of brief individual counseling sessions based on the Treating Tobacco Use and Dependence Clinical Practice Guideline [25] among 412 adult smokers. Results from the study found that participants in the MBAT condition had a higher percentage of 7-day point percentage abstinence compared with the other two groups, but the difference was not statistically significant (32.5% in UC, 39.1% in CBT, and 42.1% in MBAT at 4 weeks post quit day). Among participants classified as still smoking at final treatment sessions, recovery of abstinence was examined. The MBAT condition had significantly higher recovery rates after relapse than both the CBT and UC groups 1 week following the end of treatment (13.2% in UC, 7.0% in CBT, and 26.8% in MBAT) and 26 weeks post-quit day (0% in UC, 3.5% in CBT, and 7.1% in MBAT).

Additional data collected during the parent study described above examined the cognitive and affective mechanisms underlying MBAT versus CBT and UC for smoking cessation, including: positive and negative affect, dependence, withdrawal, craving, agency, and subjective bias towards cigarettes [26]. Spears and colleagues found that members in the MBAT group perceived greater volitional control over smoking and lower volatility of anger than CBT and UC. Although the mediators of the effect of MBAT versus CBT on lapse recovery were not observable because of small subsamples, greater perceived volitional control over smoking and lower affective volatility could be mechanisms explaining why MBAT may be a better option than both CBT and UC for promoting lapse recovery in smokers. There were no other significant differences between MBAT and CBT, nor were there significant indirect effects of MBAT versus CBT, suggesting that mindfulness and cognitive-behavioral approaches may similarly influence several of the psychosocial mechanisms implicated in tobacco dependence.

### Mindfulness training for smoking cessation (MTS)

#### Overview of protocol

Mindfulness Training for Smoking Cessation (MTS) is built upon the foundational ideas outlined in both MBSR and MBRP, but was tailored specifically for individuals who are trying to stop smoking [27]. The eight-week protocol is broken down into eight, 90-min group sessions. The primary aims of the MTS course are to (1) teach participants the skills of observing thoughts, feelings, and sensations as they are occurring in the present moment, and (2) hone the ability to accept and allow the current situation to be just as it is [16].

Sessions one and two introduce participants to how smoking has become a learned behavior through habituation and associative learning [27]. The first session introduces clients to the visceral feelings of craving, and how the urge to smoke manifests itself in the body. Session two presents the primary tool that participants can use during times of craving. The acronym RAIN (Recognize, Accept, Investigate, and Note) is used as an easy-to-remember mnemonic that clients can use when feelings of craving arise [27]. Session three introduces a traditional meditation practice, loving-kindness (metta meditation), where clients direct well wishes towards oneself and others. This practice is introduced to help clients engage with stress and negative feelings with an empathetic mind, and a kind acceptance of those feelings. Session four is the most unique aspect of the MTS course, and this is the session that sets it apart from many other MBIs, in that it encourages a formal declaration of abstinence. This session is aptly named “quit day,” and clients are asked to commit to not smoking, and are given techniques to build upon the skills of noticing the sensations that are arising in the body, primarily craving, during initial abstinence from smoking [16]. Sessions 5–7 introduce participants to how specific triggers can hinder long-term abstinence and how mindfulness practices (mindfulness of breath, RAIN, and mindful walking) can be integrated into one’s daily life to help them identify triggers and avoid smoking. Session eight summarizes all of the tools and techniques learned throughout the program and explores ways of maintaining mindful awareness in the future.

The feasibility and effectiveness of MTS was initially tested in 2013 with 55 young adult smokers [16]. This small scale pilot study found that point prevalent smoking abstinence was higher in MTS than controls, though the difference was not significant. The promising, yet underpowered results of this study have encouraged larger-scale research on the effects of the MTS protocol.

#### Literature review

An RCT conducted by Kober and colleagues compared the effects of MTS on smoking cessation with the American Lung Association’s Freedom from Smoking (FFS) program [16]. More specifically, they looked at whether stress reactivity, including neural activity during stressful scripts, related to smoking cessation directly after treatment as well as at the 3-month post-treatment follow-up. The sample included 23 smokers between the ages of 18–60 who smoked more than 10 cigarettes per day. Both treatments reduced smoking, but the MTS group demonstrated a greater rate of reduction in cigarette use during treatment, which was maintained during the 3-month post-treatment follow-up. The MTS group showed a trend toward greater 1-week point prevalence abstinence at the end of treatment (55% vs. 23%), and this difference became statistically significant at the 17-week follow-up (44% vs. 7%). Across all participants, neural activity during the stressful scenarios in the bilateral amygdala, anterior insula, mid insula, hippocampus, parahippocampal gyrus, thalamus, middle occipital gyrus, midbrain, cerebellum, and right posterior insula, as well as a second region spanning the midline across cuneus/precuneus and posterior cingulate cortex, were correlated with less reduction in cigarettes smoked per day at post-treatment [16]. During stressful scenarios, participants in the FFS group (vs. the MTS group) exhibited increased neural reactivity in several brain regions including the left amygdala, anterior, middle, and posterior insula, and bilateral portions of parahippocampal gyrus and hippocampus, putamen, thalamus, midbrain and cerebellum [16]. The MTS group did not show greater neural reactivity in any region during the stressful scenarios.

### Moment-by-moment in Women’s recovery (MMWR)

#### Overview of protocol

Moment-by-Moment in Women’s Recovery (MMWR) was developed as an adaptation of MBSR, specifically designed for low income, racially, and ethnically diverse women currently enrolled in residential SUD treatment [28]. MMWR is intended to be administered in a residential setting, a feature that distinguishes it from other MBIs [28].

MMWR is comprised of eight, 90-min group sessions, and one four-hour intensive retreat day. The first session is an introduction to mindfulness, including the definition of mindfulness and how mindfulness can play an integral role in the recovery process [29]. Sessions two and three are focused on demonstrating the automatic and constant nature of thoughts, feelings, and emotions that arise throughout the day. The theme for session three is geared at empowering clients, and showing them that the perception of their environment can dictate how a situation appears (i.e. positive or negative).

Sessions 4–6 are the core lessons dedicated to learning and practicing mindfulness skills. Session four is devoted to understanding the role that negative emotions can play in relapse [29]. The emotions of anxiety, fear, and panic are the focus of this session, and clients are asked to engage in experiential exercises to practice the skill of mindfulness during these uncomfortable situations. Session five is focused on the emotions of guilt and shame [29], which often play a role in relapse. Clients are guided through a visualization exercise to practice using mindfulness while experiencing these emotions. Practicing these skills while in a safe environment helps to give clients confidence in their new skills, and helps to bolster their confidence in their ability to prevent relapse. Session six is dedicated to using mindfulness while interacting with others, including mindful communication and the importance of positive self-talk [29].

Session seven is a four-hour “retreat” day that allows clients to immerse themselves in silent meditation to practice the skills they have been learning throughout the course. Session eight is aimed at teaching participants how to engage mindfully with anger and violence. The emotion of anger is the focus of this session, and participants are asked to identify and observe their own triggers for anger. In the final session, all of the activities and skills that were presented during the course are reviewed to solidify and sustain the concepts learned.

#### Literature review

The MMWR intervention has a very specific and niche target population that lends itself well to researching the effects of the intervention without many confounding variables. In a large-scale RCT, Black and colleagues tested the efficacy of MMWR on improving SUD treatment retention when applied as an adjunctive intervention to residential treatment compared with a neurobiology of addiction psychoeducation course (control condition) [17]. The sample was comprised of 200 adult women diagnosed with SUD and admitted to the residential SUD treatment program study site. The primary outcomes of the study were discharge status (if a client developed the necessary skills to complete residential treatment), days until discharge (the number of days a client remains in residential treatment beginning with the first day of the intervention and ending at day 150), and self-report measures of mindfulness disposition, perceived stress, distress tolerance, emotion regulation, psychological distress, affect, and drug and alcohol craving [17]. The count of women defined as treatment completers (client completed SUD treatment) was 43/100 in the MMWR group versus 31/100 in the control group. The count of women in-residence (client still at the residential treatment site at day 150) was 15/100 in the MMWR group and 27/100 in the control group. Among the 84 treatment non-completers (client left the facility before the end of treatment), satisfactory progress was made by 16/42 women in the MMWR group and 10/42 women in the control group. However, there were no differences between conditions for any of the self-report measures, including craving.

## Conclusions

The purpose of this manuscript was to provide a session-by-session overview of treatment protocols of MBIs and to systematically review randomized controlled trials (RCTs) of MBIs for SUD, with a focus on studies published after 2017 to update a previous systematic review conducted by Li et al. [19]. Key findings on clinical implications, current limitations in the field, and suggestions for the road ahead are presented below.

There is a variety of MBIs in the field of SUD treatment. All of the protocols we reviewed have some commonalities that target factors maintaining SUD. Bringing one’s attention to the immediate experience of the present moment is the foundation upon which all of these interventions are based. Regardless of the techniques employed, the goal of mindfulness is to stop (momentarily) rumination about past events and the planning of future actions. Most of the interventions we reviewed have both formal and informal meditation practices that are designed to encourage participants to break the cycle of thoughts and stop the process of acting on autopilot. One of the key components of SUD is a desire (i.e. craving) to engage in substance use due to habit or a desire to increase momentary enjoyment or avoid discomfort. One of the primary aims of the MBIs we reviewed is bringing focused attention to whatever is arising in the present moment (i.e. craving, sadness, pain, etc.) without judgment, and without a need to engage with, including actively avoiding, those feelings.

Despite sharing some commonalities, many of these programs have been designed for different populations and substances. One of the cornerstones of MBRP is becoming aware of triggers and finding ways to cope with cravings. This focus on triggers and craving may suggest that MBRP is most beneficial for an individual in the earlier stages of changing their substance use. MTS, MBAT, and MMWR all have strong foundational links to MBSR. These three protocols are primarily centered around bringing attention to the constant stream of thoughts everyone has during the day. This observation of thoughts is designed to cut through the automaticity of SUD. MABT is an intervention centered around interoception and becoming aware of the deleterious effects of engaging in addictive behavior. This intervention can be beneficial at teaching participants self-care practices to take the place of substance use. The different focal points that these protocols have may suggest that they are better suited for later stages of substance use behavior change. Future research should focus on these differing focal points to determine the best time in the recovery process to deliver these differing MBIs.

The growing field of MBIs for SUD has produced mixed results. While some studies have yielded positive outcomes, others have found that MBIs perform no better than existing treatments or control conditions. Some of the findings from recent MBRP research show that this protocol may be quite effective for individuals with high SUD severity *or* high levels of co-occurring anxiety and depression symptoms (e.g., [31, 35]). Several reviewed studies also found promising effects of MBIs on a range of psychiatric outcomes, including depression and anxiety symptoms (e.g., [26, 35, 44]), psychological flexibility (e.g., [40]), posttraumatic stress disorder symptoms, and positive affect (e.g., [15]). With the high prevalence of SUD comorbid with other mental health conditions, positive results in populations with high levels of depression and anxiety symptoms, as well as decreases in psychiatric distress over MBIs, are encouraging. However, not all studies examining secondary psychiatric distress outcomes evidenced benefits of MBIs in comparison to control groups (e.g., [41]), and therefore additional research is needed to determine particular components of MBIs and/or the dose of mindfulness practice that best targets psychiatric distress.

Another positive outcome we found was the beneficial effects that MBIs have on cue-reactivity and attentional bias [47, 48, 52]. Mitigating the sustained, motivated focus on substance-related cues, as well as an individual’s reaction to these cues, may prove to be one of the most beneficial aspects of MBIs in the treatment of SUD. An additional factor that MBIs appear to target is perceived stress. Many of the protocols we reviewed demonstrated that participants had more volitional control of stressful situations that may have previously led them to engage in substance use [23, 32, 33]. These findings could be linked to the process of non-judgmental awareness that all of the protocols encourage.

Despite some of the more positive findings, some of the articles we reviewed showed no significant differences between MBIs and other evidenced-based treatments. Many studies found little to no effect on secondary outcomes such as positive and negative affect, emotion regulation, or mindfulness scores [23, 36, 41]. This lack of improvement in putative mechanisms of change may be due to the fact these are not the right mechanisms to be investigating. With the large number of positive outcomes that many of these studies did find, it is hard to imagine that these MBIs are not positively impacting behavior change on some level. One challenge in interpreting outcomes from MBIs is the varying definitions of mindfulness that have been utilized, both as an outcome and as content in MBIs. Therefore, identifying the most appropriate outcomes and mediators to examine is difficult when there is such a broad spectrum of definitions for the behavioral mechanisms targeted by MBIs. Future research must focus on what underlying behaviors mindfulness targets to develop and use appropriate evaluation tools and better fidelity measures to determine the effects of these interventions. Hopefully, future research may be able to shed some light on the best mechanisms of change to be investigated.

However, we found several limitations that make it difficult to draw firm conclusions. First, there is a pressing need for large scale RCTs with equivalent control conditions and long-term follow-ups. Many of the existing studies have small sample sizes with limited generalizability, lack of equivalent time/effort controls, and the results of the studies have small effect sizes with large confidence intervals. Another factor that needs to be controlled for is treatment fidelity. With the ability to administer certain MBIs after having simply bought the treatment manual or taken a weekend course, there is a need for uniformity when administering these interventions. Finally, one of the biggest questions yet to be answered in the study of MBIs is the minimum effective dose required to exhibit the desired behavior change. Many of the current MBIs follow the structure of the original MBSR protocol developed by Kabat-Zinn in the early 1980s with little variation. The entire field of mindfulness research would benefit from testing variations from the standard 8-week intervention initially developed to determine the appropriate dose-response of meditation in these clinical populations. We believe that meditation is a dose-dependent intervention, but future research must determine the minimum threshold (i.e. time dedicated to meditation in both formal and informal practice) to see the expected improvements in psychiatric, cognitive, and substance use outcomes. Conducting large RCTs with stringent experimental controls may help provide a clearer picture of the effectiveness of MBIs.

Results of the present review are also qualified by several limitations. We limited ourselves to the search terms utilized in a previous review by Li and colleagues [19], and therefore may have overlooked relevant articles that were not captured by these search terms. Similarly, we chose to focus on SUD, specifically, as opposed to addictive behaviors, broadly. Examining MBIs for behavioral addictions is an emerging area of research and future reviews of these studies may provide further insight into the mechanisms of MBIs for addictive behaviors. Lastly, the results of the present review were purely based on a qualitative, not quantitative, analysis. Although we chose to provide a narrative review of the efficacy of MBIs for SUD, best practices for these interventions, and future research directions (including future meta-analyses) in this area will continue to provide critical information for understanding the effectiveness of MBIs for addictive behaviors.

Mindfulness has been used for centuries to help with the fleeting and sometimes difficult nature of the human mind, and these practices have recently proliferated in Western medicine, particularly to mitigate mental health disorders, including SUD. A majority of the MBIs for SUD reviewed use similar foundational concepts to help clients bring focused attention to their daily lives, indicating that the aspects of mindfulness are likely viable interventions for SUD. Continued research, including large-scale RCTs with equivalent control groups and long-term follow-ups, will clarify the function mindfulness can play in the treatment of SUD, and will also help develop the most effective way to integrate mindfulness into existing SUD treatment programs. Widespread interest in mindfulness and its benefits in the clinical setting continue to proliferate, and results from future research will show us the best contexts and settings for its use. Further, given evidence that MBIs are at least as effective, and in some cases more effective, than existing treatments would suggest that these interventions are ready for widespread dissemination. Future research should focus on approaches to increase the dissemination and implementation of MBIs into more community treatment settings [61]. Cost-effectiveness analyses of MBIs for SUD would also be an important next step to inform policy makers and tax payers. It is potentially the case that investing in more SUD treatment programs that offer MBIs will potentially yield cost savings, as has been shown for other MBIs [62].

## Data Availability

Not applicable.

## Abbreviations

AAQ: Acceptance and Action Questionnaire
AAQ-SA: Acceptance and Action Questionnaire – Substance Abuse
AB: Attentional Bias
ACT: Acceptance and Commitment Therapy
AIPQ: Affective Information Processing Questionnaire
ASI: Addiction Severity Index
BAI: Beck Anxiety Inventory
BDI: Beck Depression Inventory
BIS: Barratt Impulsiveness Scale
BPI: Brief Pain Inventory
CBT: Cognitive Behavioral Therapy
CES-D: Center for Epidemiologic Studies Depression Scale
CM: Contingency Management
COMM: Current Opioid Misuse Measure
DASS: Depression Anxiety Stress Scales
DBT: Dialectical Behavior Therapy
DERS: Difficulties in Emotion Regulation Scale
DrInC: Drinker Inventory of Consequences
DUD: Drug Use Days
EEG: Electrocardiography
EMA: Ecological Momentary Assessment
FFMQ: Five Facet Mindfulness Questionnaire
FFMQ-BR: Five Facet Mindfulness Questionnaire-Brazil
FFS: Freedom from Smoking
FIM: Functional Independence Measure
fMRI: Functional Magnetic Resonance Imaging
GAIN: Global Appraisal of Individual Needs
HAD: Hospital Anxiety and Depression
HCQ: Heroin Craving Questionnaire
HDD: Heavy Drinking Days
HE: Health Education
HR: Heart Rate
HRV: Heart Rate Variability
HSI: Heaviness of Smoking Index
IRR: Incidence Rate Ratio
ITT: Intent To Treat
LPP: Late Positive Potential
MAAS: Mindful Attention Awareness Scale
MAIA: Multi-dimensional Assessment of Interoceptive Awareness
MABT: Mindful Awareness in Body-Oriented Therapy
MBAT: Mindfulness-Based Addiction Treatment
MBCT: Mindfulness-Based Cognitive Therapy
MBI: Mindfulness-Based Intervention
MBRP: Mindfulness-Based Relapse Prevention
MBSR: Mindfulness-Based Stress Reduction
MLQ: Meaning in Life Questionnaire
MMT: Methadone Maintenance Therapy
MMWR: Moment-by-Moment in Women’s Recovery
MORE: Mindfulness Oriented Recovery Enhancement
MTS: Mindfulness Training for Smokers
NADA: Non-dual Awareness Dimensional Assessment
OUD: Opioid Use Disorder
PACS: Penn Alcohol/Drug Craving Scale
PANAS: Positive and Negative Affect Schedule
PASS: Pain Anxiety Symptoms Scale
PCL: PTSD Checklist
PSS: Perceived Stress Scale
PSS-SR: PTSD Symptom Scale – Self-Report
PTSD: Post-traumatic Stress Disorder
QSU: Questionnaire of Smoking Urges
RAIN: Recognize, Accept, Investigate, and Note
RCT: Randomized Controlled Trial
RSA: Respiratory Sinus Arrhythmia
SBQ: Subjective Attentional Bias Questionnaire
SDS: Severity and Dependence Scale
SFS: Substance Frequency Scale
SES: Smoking Self-Efficacy Scale
SG: Support Group
ST: Standard Treatment
SUD: Substance Use Disorder
TAU: Treatment as Usual
tDCS: Transcranial Direct Current Stimulation
TLFB: Timeline Followback
UC: Usual Care
UPPS-P: Urgency, Premeditation, Perseverance, Sensation Seeking, and Positive Urgency
WBSI: The White Bear Suppression Inventory
WHE: Women’s Health Education
WISDM: Wisconsin Inventory of Smoking Dependence Motives
WSWS: Wisconsin Smoking Withdrawal Scale
